# Supplementary material for: Safety and efficacy of an artificial intelligence-enabled decision tool for treatment decisions in neovascular age-related macular degeneration and an exploration of clinical pathway integration and implementation: protocol for a multi-methods validation study
Source: BMJ Open. 2023 Feb 1;13(2):e069443. doi: 10.1136/bmjopen-2022-069443 (PMC9896175; doi:10.1136/bmjopen-2022-069443)
Supplement: Supplementary data [file bmjopen-2022-069443supp001.pdf]

Systematic sampling strategy

Step	Question	Action if… Yes	Action if… No
**1**	Does this eye have no retinal diagnosis beside AMD or is it enrolled in a study?	Go to step 2	Reject this patient
**2**	Is this visit more than 10 weeks after the eye's first IVI?	Go to step 3	Switch to the next visit, go to step 2
**3**	Does this visit involve anti-VEGF treatment for nAMD?	Go to step 4	Switch to the next visit, go to step 2
**4**	Is this visit conducted under the loading or TEX protocols?	Go to step 5	Switch to the next visit, go to step 2
**5**	Are the VAs of interest free from the influence of other interventions?	Go to step 6	Switch to the next visit, go to step 2
**6**	Does this visit have an accompanying consultation recorded?	Go to step 7	Switch to the next visit, go to step 2
**7**	Is the treatment interval stated?	Go to step 8	Switch to the next visit, go to step 2
**8**	Is there a VA available for this visit and the prior?	Go to step 9	Switch to the next visit, go to step 2
**9**	Are there co-located 25 slice fovea-centred OCTs available for this visit and the prior?	Collect data	Switch to the next visit, go to step 2

AMD = Age-related macular degeneration, IVI = Intravitreal Injection, VEGF = Vascular Endothelial Growth Factor, nAMD = neovascular Age-related macular degeneration, TEX = Treat and Extend, VA = Visual Acuity, OCT = Optical Coherence Tomography
