# Supplementary material for: Safety and efficacy of an artificial intelligence-enabled decision tool for treatment decisions in neovascular age-related macular degeneration and an exploration of clinical pathway integration and implementation: protocol for a multi-methods validation study
Source: BMJ Open. 2023 Feb 1;13(2):e069443. doi: 10.1136/bmjopen-2022-069443 (PMC9896175; doi:10.1136/bmjopen-2022-069443)
Supplement: Author's manuscript [file bmjopen-2022-069443.draft_revisions.pdf]

BMJ Open is committed to open peer review. As part of this commitment we make the peer review history of every article we publish publicly available.

When an article is published we post the peer reviewers' comments and the authors' responses online. We also post the versions of the paper that were used during peer review. These are the versions that the peer review comments apply to.

The versions of the paper that follow are the versions that were submitted during the peer review process. They are not the versions of record or the final published versions. They should not be cited or distributed as the published version of this manuscript.

BMJ Open is an open access journal and the full, final, typeset and author-corrected version of record of the manuscript is available on our site with no access controls, subscription charges or pay-per-view fees (http://bmjopen.bmj.com).

If you have any questions on BMJ Open's open peer review process please email info.bmjopen@bmj.com

BMJ Open

A multi-methods validation of artificial intelligence-enabled macula services: a protocol

Journal:	*BMJ Open*
Manuscript ID	bmjopen-2022-069443
Article Type:	Protocol
Date Submitted by the Author:	21-Oct-2022
Complete List of Authors:	Hogg, Henry; University of Newcastle upon Tyne; Newcastle Upon Tyne Hospitals NHS Foundation Trust<br>Brittain, Katie; University of Newcastle upon Tyne<br>Teare, Dawn; University of Newcastle upon Tyne<br>Talks, S.; Newcastle Upon Tyne Hospitals NHS Foundation Trust<br>Balaskas, Konstantinos; Moorfields Eye Hospital NHS Foundation Trust; University College London Institute of Ophthalmology<br>Keane, Pearse ; University College London Institute of Ophthalmology; Moorfields Eye Hospital City Road Campus<br>Maniatopoulos, Gregory; Northumbria University; Newcastle University Faculty of Medical Sciences
Keywords:	QUALITATIVE RESEARCH, Medical retina < OPHTHALMOLOGY, Organisation of health services < HEALTH SERVICES ADMINISTRATION & MANAGEMENT, Health informatics < BIOTECHNOLOGY & BIOINFORMATICS, STATISTICS & RESEARCH METHODS

SCHOLARONE™
Manuscripts

A multi-methods validation of artificial intelligence-enabled macula services: a protocol

Short title: AI-enabled macular service validation

Authors: Dr H.D. Jeffry Hogg[1,2,3]

Prof K. Brittain[1]

Prof D. Teare[1]

Mr. S.J. Talks[2]

Mr. K. Balaskas[3,4]

Prof P.A. Keane[3,4]

Dr G. Maniatopoulos[5,1]

Institutions: 1. The University of Newcastle upon Tyne, Newcastle upon Tyne, Tyne and Wear, NE1 7RU, U.K.

2. The Newcastle upon Tyne Hospitals NHS Foundation Trust, Newcastle upon Tyne, Tyne and Wear, NE1 7RU, U.K.

3. Moorfields Eye Hospital NHS Foundation Trust, 162 City Road, London, EC1V 2PD, U.K.

4. University College London Institute of Ophthalmology, 11-43 Bath Street, London, EC1V 9EL, U.K.

5. Northumbria University, Sutherland Building, 2 Ellison Place, Newcastle upon Tyne, NE1 8ST, U.K.

For peer review only - http://bmjopen.bmj.com/site/about/guidelines.xhtml

Corresponding: Jeffry Hogg, Newcastle Eye Centre, Royal Victoria Infirmary, Queen Victoria Rd, Newcastle upon Tyne, Tyne and Wear, NE1 4LP, U.K. Tel: 07900603591, Fax: 01912826269, Email: Jeffry.hogg@ncl.ac.uk

Financial support: This work is funded entirely by a National Institute of Health Research Doctoral Fellowship award; NIHR301467

Conflicts of interest: Google Health™ and Deepmind ™ are supporting this project through access to their analytical software at no charge.

For peer review only

For peer review only - http://bmjopen.bmj.com/site/about/guidelines.xhtml

Abstract

Introduction

The management of neovascular age-related macular degeneration (nAMD) is one of the largest single-disease contributors to NHS hospital outpatient appointments. The clinician time nAMD demands could be reduced through the partial automation of treatment decisions. Established artificial intelligence (AI)-enabled tools, capable of segmenting different tissue types on retinal imaging, could identify the disease activity or stability which direct these treatment decisions, but they have only undergone pre-clinical validation for adjacent use-cases. A primary qualitative investigation of how stakeholders in nAMD care would perceive such an AI-enabled decision tool is also absent. This multi-methods study aims to establish the safety and efficacy of an AI-enabled decision tool to make treatment decisions for nAMD and understand where on the clinical pathway it could sit and what factors are likely to influence its implementation.

Methods and analysis

Retrospective imaging and clinical data will be collected from nAMD clinic visits at a single NHS ophthalmology service, including judgements of nAMD disease stability or activity made in real-world consultant-led-care. An AI-enabled retinal segmentation tool and a rule-based decision tree will independently analyse imaging data to report nAMD stability or activity for each of these clinic visits. Independently, an external reading centre will receive both clinical and imaging data to generate an enhanced reference standard for nAMD stability or activity for each clinic visit. The non-inferiority of the relative negative predictive value of AI-enabled reports on disease activity relative to consultant-led-care judgements will then be tested. In parallel, approximately 40 semi-structured interviews will be conducted with key

For peer review only - http://bmjopen.bmj.com/site/about/guidelines.xhtml

nAMD service stakeholders, including patients. Transcripts will be coded using a theoretical framework and thematic analysis will follow.

Ethics and dissemination

NHS Research Ethics Committee and UK Health Research Authority approvals are in place (21/NW/0138). Written and oral dissemination is planned to public, clinical, academic and commercial stakeholders.

Keywords:

Age-related macular degeneration, non-inferiority study, AI validation, qualitative research, semi-structure interviews, implementation

Strengths and limitations of this study

- This non-inferiority study examines an AI use-case supported by patients and the public, with scope for improving the efficiency with which limited human resources are applied across the NHS and elsewhere.
- This AI use-case has a high degree of explainability as it relies on reviewable tissue segmentation and a simple rule-based decision tree which mimic widely used treatment paradigms.
- The multi-methods approach allows insights beyond efficacy alone to consider the effectiveness, mechanisms and system impacts of this complex intervention.
- Due to the pre-clinical nature of this study, stakeholder perspectives concerning this AI-use case will be hypothetical rather than from direct experience.

For peer review only

Introduction

A threat to the efficacy of UK National Health Service (NHS) ophthalmology services is posed by insufficient ophthalmologist availability, in the face of a growing clinical need.[1, 2] Ophthalmology continued to incur more clinic appointments than any other NHS specialty through the Covid-19 pandemic, with age related macular degeneration (AMD) as the most common cause for these appointments.[2] AMD is the leading cause for certification of visual impairment in the UK and affects just over a quarter of Europeans aged over 60 to some extent.[3, 4] Only the disease course of a severe form of AMD, complicated by choroidal neovascularisation (nAMD), can benefit from current treatment and is thought to affect 1.4% of Europeans aged over 60.[3] Real-world data from a NHS centre showed that even before the impact of the Covid-19 pandemic, delay in delivering planned treatments for nAMD was common and associated with sight loss for the patients concerned.[5] This same NHS centre had recently displayed better nAMD visual outcomes than a mean of 12 large UK centres, suggesting the observed treatment delays are likely to be representative of the whole nation.[6] Since these observations, the Covid-19 pandemic has introduced additional nAMD treatment delays and avoidable sight loss, further developing the urgent need to augment clinical capacity for nAMD treatment.[7]

Clinical artificial intelligence (AI) is an increasing target of investment from government, industry and academia, often with the hope of increasing service capacity independent of the limits posed by clinician availability.[8-10] Ophthalmology is one of the most heavily researched specialties in AI medical image analysis with the non-contact infra-red based imaging modality, optical coherence tomography (OCT), accounting for much of this.[11] Retinal OCT scans form the substrate for several clinical AI tools, which anatomically segment the images to support various clinical decisions including triage and diagnosis.[12] The impact of these clinical AI functions on the efficacy and efficiency of primary to

For peer review only - http://bmjopen.bmj.com/site/about/guidelines.xhtml

For peer review only

secondary care referrals in ophthalmology are the subject of an on-going prospective clinical studies.[13, 14] However, an even greater cumulative demand on ophthalmologists' time is making decisions about the treatment of nAMD within secondary care. Treatment takes the form of intravitreal injections (IVIs) of anti-vascular endothelial growth factor (anti-VEGF) agents which are recommended for variable periods of time and at varying intervals depending on changes in the retina observed through OCT.[15, 16] Whilst the decisions about treatments often involve ophthalmologists, the injections themselves are increasingly performed independently by specialised nurse practitioners and allied health professionals.[17, 18] Given the highly protocolised, OCT-dependent, nAMD treatment pathway and pre-existent clinical AI capable of independently quantifying the OCT features which inform that treatment, there is a clear opportunity for AI-enabled nAMD services to expand capacity and reduce avoidable real-world sight loss.[12, 16]

Despite this tension for change, clinical AI is yet to be implemented within routine NHS ophthalmic practice. This is at least partly due to the lack of evidence to forecast the effectiveness of clinical AI in the real-world setting and how it will interact with the various stakeholders and contexts which must host the technology.[19] Alongside other innovations such as portable OCT technology and community outreach treatment services, AI may enable even greater changes to the way in which macular services are delivered (Figure 1).[20, 21] The complexity derived from these interdependent technologies, contexts and stakeholders must be better understood if implementation is to optimise patient benefit from AI-enabled nAMD services. This single-centre multi-methods study aims to generate the necessary evidence by an external validation of the technology with real-world data and exploring the interdependent factors which will influence the implementation of AI-enabled nAMD services. In doing so, we aim to move beyond efficacy to consider intervention effectiveness and construct a theoretical basis to guide future implementation strategies.[22]

For peer review only - http://bmjopen.bmj.com/site/about/guidelines.xhtml

A quantitative study will be performed to test the non-inferiority of AI-enabled reports of nAMD disease activity against judgements made in consultant-led-care. In addition, a qualitative study based on semi-structured interviews with key stakeholders and patients will aim to explore where the clinical AI could sit within the nAMD care pathway and the factors likely to influence its implementation.

Methods and analysis

Quantitative methods

Sampling method

The electronic medical record (EMR) at Newcastle upon Tyne Hospitals NHS Foundation Trust (NuTH) will be searched to identify clinic visits where individuals received anti-VEGF IVI to treat nAMD. Patients at NuTH are treated under three different regimens dependent upon the length of their diagnosis and joint decisions between clinicians and patients, loading, treat-and-extend and pro-re-nata (Panel 1).

For peer review only - http://bmjopen.bmj.com/site/about/guidelines.xhtml

Loading protocol: Starts at diagnosis of nAMD and consists of three anti-VEGF IVIs at four-week intervals (visits two and three do not include consultation or imaging), followed by IVIs at eight-week intervals, or less if signs of disease activity persist, for the remainder of the year.

Treat-and-extend (TEX) protocol: Starts one year after treatment initiation and dictates that the interval of anti-VEGF IVIs is increased in two-week increments until evidence of disease activity is noted at which point the treatment interval is reduced. If extension beyond a certain interval is noted to result in observable disease activity a (unspecified) number of times, then that interval ceases to be modified.

Pro-re-nata (PRN) protocol: Initiated as a joint decision for patients who appear to have little or no disease activity having been on one of the other two protocols. Here it is not assumed that an IVI will be given at each review, but only if evidence of disease activity is noted. The observation of returning disease activity may also lead to the return to one of the other two protocols.

Panel 1. Summary of neovascular age-related macular degeneration (nAMD) treatment at Newcastle upon Tyne Hospitals NHS Foundation Trust. IVI=Intravitreal Injection, VEGF = Vascular Endothelial Growth Factor

Eligible visits from this dataset of 70,884 will be randomly selected for manual review of patient EMR files by a clinician practicing at NuTH (HDJH) to screen against eligibility criteria (Panel 2). These criteria exclude clinical visits that took place before current treatment and OCT imaging protocols were established or after treatment decisions may have been influenced by the Covid-19 pandemic. They also exclude visits conducted under the PRN treatment protocol as all visits on this protocol meeting the inclusion criterion for a same day anti-VEGF IVI must have been judged by the clinician to show disease activity and

For peer review only - http://bmjopen.bmj.com/site/about/guidelines.xhtml

will therefore not be representative of PRN clinic visits generally. To systematically apply and record these eligibility criteria an a-priori screening approach was developed and trialled with the clinicians (HDJH, SJT, PAK, KB) and biostatistician (DT) in the research team (Supplementary file 1).

Inclusion criteria

- **Eye diagnosed with nAMD**
- **One or more prior anti-VEGF IVI at NuTH**
- OCT imaging available for both the included and prior visits
- Clinic visit note states intended IVI interval
- Clinic visit included same-day IVI

Exclusion criteria

- **Retinal diagnosis other than nAMD in included eye**
- Visit before 2016
- Visit during or after March 2020
- Visits conducted under the pro-re-nata treatment protocol

Panel 2. Eligibility criteria for patients (in bold) and clinic visits. nAMD = Neovascular age-related macular degeneration, IVI = Intravitreal injection, NuTH = Newcastle upon Tyne Hospitals, OCT = Optical coherence tomography

Data collection and processing

For each included clinic visit the following data will be recorded to characterise the dataset:

- Anonym for the individual
- Eye laterality

For peer review only - http://bmjopen.bmj.com/site/about/guidelines.xhtml

- Gender
- Self-reported ethnicity
- Home address postal code stem
- Individual's age at that visit

The following will be recorded to send anonymised to Moorfields Reading Centre to generate a report of disease stability or activity to act as an enhanced reference standard for each visit. This information will also facilitate more meaningful post-hoc error analysis to explore the mechanisms of failure which the AI-enabled tool may exhibit. This list was developed through additions to a proforma from a recent exemplar protocol:[23]

- OCT and visual acuity (VA) for that visit and the prior
- VA for the fellow eye at that visit
- Time since first nAMD treatment at that visit
- Total IVIs for nAMD in that eye up until that visit
- Observed interval since that eye's last IVI
- Presence or absence of evolving macular haemorrhage being recorded
- Time since increasing disease activity was last observed
- Treatment interval associated with that observation

The following data will also be recorded from each consultant-led-care visit to assess the primary and secondary outcomes and will not be sent to Moorfields Reading Centre:

- Judgement of disease activity or stability
- Planned interval to next IVI
- Professional group conducting the consultation
- Treatment protocol the visit was conducted under (Panel 1)

For peer review only - http://bmjopen.bmj.com/site/about/guidelines.xhtml

Separately, the present and prior pairs of OCT images relating to the same clinic visits will be transferred to Moorfields Eye Hospital NHS Foundation Trust for AI-enabled retinal segmentation.[12] The differences in retinal tissue volumes will be used in a rule-based decision tree to produce an AI-enabled binary report of disease activity or stability for each included visit.

AI-enabled decision tool

The intervention to be tested on retrospective OCT imaging data is a deep learning tool with a U-net architecture with previously published details of training and validation.[12] It can produce volume quantification for the neurosensory retina, retinal pigment epithelium, fibrovascular pigment epithelium detachment, drusenoid pigment epithelium detachment, subretinal hyper-reflective material, subretinal fluid, intraretinal fluid, posterior hyaloid, epiretinal membrane and serous pigment epithelium detachment. The volumes of neurosensory retina, subretinal fluid and intraretinal fluid will be compared for the retinal OCT at each included visit and the related prior visit. The exact decision tree threshold for inter-visit changes in each of these tissue groups will be developed using pilot data, but an initial approach of reporting disease stability for all cases except those where any of the aforementioned tissue volumes increases >5% between the current and prior visit is planned.[24] This binary output was preferred over a scalar recommendation of treatment interval to preserve the tools' value across different treatment protocols (Panel 1).

Outcomes measures

Over-treatment marginally increases the cumulative risk of IVI complications and the cost to the provider, but justifiably the main concern of patients and carers consulted in designing this study was sight loss through under-treatment.[5] Consequently, the probability of AI-enabled reports of disease stability being correct relative to judgements made in real-world consultant-led care has been taken as the most clinically relevant measure of diagnostic

For peer review only - http://bmjopen.bmj.com/site/about/guidelines.xhtml

accuracy. This has led to a non-inferiority design with the relative negative predictive value (NPV) as the primary outcome.

Secondary outcomes will be:

- A comparison of other standard diagnostic accuracy metrics between AI-enabled reports of disease activity and judgements from consultant-led-care accompanied by confusion matrices (Table 1)
- A comparison of the diagnostic accuracy of the five healthcare professional groups conducting consultations in consultant-led-care (nurses, optometrists, ophthalmology specialty trainees, medical retina sub-specialty fellows, medical retina sub-specialty consultants)
- A comparison of the treatment intervals recommended in real-world consultant-led care and the treatment intervals that would be derived from AI-enabled reports of disease activity given the treatment protocol
- A case-by-case exploration of false-positive and false-negative reports of disease activity from the AI-enabled decision tool and consultant-led care

<u>Justification of study design and sample size</u>

An enhanced reference standard is required to facilitate comparison between a potential AI-enabled nAMD service and the real-world gold standard, consultant-led-care. Moorfields Reading Centre has an international reputation and track record in meeting this need for prior studies and will receive imaging and clinical data for each case in the present study to generate an enhanced reference standard.[12, 13, 23] The binary decision under examination for each included visit is whether the data suggest disease stability or activity. This simplification of the scalar number of weeks between treatments or the three-option decision regarding treatment interval maintenance, extension or reduction is more broadly relevant to

For peer review only

real-world practice where clinician and patient preferences about how treatment intervals should be altered on account of disease activity vary (Panel 1).[16] It also lowers the risk of inappropriately labelling a decision made in consultant-led-care with subtle influences from patient or clinician preference as 'incorrect'. Whether binary judgements of disease activity (positives) or stability (negatives) from consultant-led-care and the AI-enabled decision tool are labelled as true or false will be decided by the independent judgement of the Moorfields Reading Centre (Table 1).

Table 1. Template confusion matrix showing the different possible classification of Artificial Intelligence (AI)-enabled reports of disease activity and judgements from consultant-led-care (CLC) for each eligible case.

	Moorfields Reading Centre identifies disease stability (negative)	Moorfields Reading Centre identifies disease activity (positive)
AI identifies disease stability (negative)	AI True negative	AI False negative
AI identifies disease activity (positive)	AI False positive	AI True positive
CLC identifies disease stability (negative)	CLC True negative	CLC False negative
CLC identifies disease activity (positive)	CLC False positive	CLC True positive

Treatment decision data to support power calculations are unavailable for this novel use case. Considering a different use case, the deep learning model to be applied within the proposed AI-enabled decision tool has demonstrated equivalent or superior retinal diagnostic performance to consultant specialists. Relative to final real-world clinical diagnoses it produced an area under the ROC curve of 99% for retinal diseases including nAMD.[12] This performance was dependent on the same intermediate anatomical segmentation step that will form the basis for the AI-enabled decision tool for nAMD treatment proposed here. This has supported the feasibility of the current proposal but does not provide the level of certainty required to perform a robust power calculation, which requires sufficiently accurate estimates of the NPV of both judgements from consultant-led-care and AI-enabled reports from paired

For peer review only - http://bmjopen.bmj.com/site/about/guidelines.xhtml

data.[25] Therefore, a pilot dataset will be collected and sent for independent processing by Moorfields Reading Centre and the AI-enabled decision tool to supply these estimates. Prior work has established that for binary outcomes such as the one under study, little improvement is seen in estimate precision or bias by increasing the size of the pilot dataset above 100.[26] A preliminary review of 100 NuTH consultant-led-care nAMD clinic visits under loading or TEX treatment protocols found that 79 reported disease stability, and would be classed as negative cases which could contribute to estimating NPV. Given that about 79% of eligible visits are classed as negative the randomly sampled pilot dataset required to accrue 100 negative cases is expected to be around 127 cases (100 / 0.79), but the consultant-led-care judgement for each case will be reviewed as it is curated to ensure the pilot dataset contains 100 negative cases. The estimated NPV of AI-enabled reports and judgements from consultant-led-care will be derived from this pilot dataset and inform a power calculation. From this, the number of additional eligible cases required to be collected and processed by Moorfields Reading Centre and the AI-enabled decision tool to test the non-inferiority of AI-enabled report NPV relative to judgements from consultant-led-care will be established.[25] This power calculation will include a significance level $\alpha = 0.05$, a power $\beta = 0.90$ and a relative non-inferiority margin of $\delta = 0.90$. Although this non-inferiority margin is relative, it will be similar and no larger than an absolute equivalent. This allowed the application of 10% non-inferiority margins applied in comparable studies.[23, 27] In evaluating this non-inferiority margin it is helpful to remember that the least desirable outcome of a false negative would yield a two or four week delay for the next planned treatment rather than treatment cessation and that 22% of patients are estimated to experience more than four weeks of delay to treatment in a year in current consultant-led care.[5] As such the null hypothesis will be rejected if the lower confidence limit for the relative NPV of AI-enabled reports is greater than 0.90.[25] This will be visually presented with 2-sided 95% confidence

For peer review only

For peer review only - http://bmjopen.bmj.com/site/about/guidelines.xhtml

interval to explore the possibility of superiority with a threshold γ of $1/\delta$; $\gamma = 1.11$ (Figure 2). The sample size will not be powered for secondary outcomes.

Data analysis

The relative NPV of AI-enabled reports / judgements from consultant-led-care will be calculated with 95% confidence intervals to see if the inferiority, non-inferiority or superiority of AI-enabled reports of disease activity can be established (Table 1). For secondary outcomes, diagnostic accuracy statistics for each group will be reported descriptively with 95% confidence intervals for each group, along with confusion matrices. Clinical and imaging data from cases of false positives and false negatives of the AI-enabled reports will be reviewed by clinical members of the team, supported by the AI development team where necessary, to try to understand the mechanisms and potential consequences of AI-enabled decision tool failures. STARD reporting guidelines will be used alongside the STARD-AI extension if available at the time of writing.[28, 29]

Qualitative methods

Sampling

A recent systematic review outlined the importance of all stakeholder perspectives in understanding the interdependent factors that influence clinical AI implementation.[30] Despite this, the qualitative literature regarding clinical AI is dominated by healthcare professionals' perspectives and has particularly limited representation from carers.[30] Consequently, the present study will aim to explore perspective from all stakeholders. This will include patients from the NuTH nAMD clinic, carers of patients from the same clinic, doctors, nurses, photographers and optometrists working in the clinics, primary care doctors and optometrists, hospital managers, relevant industry and charity sector professionals, and care commissioners. An approximate target of 40 participants has been set, but the targeting and scale of recruitment will be refined by themes and stakeholders identified in the data.

For peer review only - http://bmjopen.bmj.com/site/about/guidelines.xhtml

Data collection

Topic guides have been informed by the aforementioned systematic review and input from clinicians, academics, patients and lay members of the study team. In their initial form these topic guides focus on:

- Stakeholders' experiences of the present macula service
- Their perceptions of clinical AI generally and specifically in recommending treatment intervals for nAMD
- Where this AI-enabled decision tool should be placed within the healthcare pathway
- Factors like to affect the implementation of this AI-enabled decision tool
- How relationships between stakeholders and with the AI itself may change or develop

The topic guides will be used flexibly to guide semi-structure interviews and continuously iterated as data are analysed in parallel. Depending upon participant preference interviews may be conducted face-to-face or remotely by telephone or teleconferencing platforms. Interviews will be recorded and transcribed for text-based coding and analysis. Interviews will be delivered by the same researcher (HDJH) throughout, who is an ophthalmologist with lived experience of working in NuTH nAMD clinics and prior training and experience in qualitative research methods. Although patients with prior clinical contact with the interviewer will be ineligible for recruitment and the interviewer will be presented as a researcher rather than a clinician, their status as a healthcare professional may emerge unintentionally and could influence data elicitation.[31] It is also true that some of the healthcare professional interviewees will have worked with the interviewer as a colleague in the past which is likely to influence data. Reflective journaling will be used to transparently report and manage these influences on data collection, analysis and interpretation.[32] Data will also be reviewed throughout the collection period by the wider study team, including lay

For peer review only - http://bmjopen.bmj.com/site/about/guidelines.xhtml

representatives, to help identify and manage biases in sampling, data collection, analysis and interpretation.

Data analysis

The same researcher (HDJH) will familiarise themselves with data as it is collected and iteratively place data into a-priori codes and categories derived from a qualitative framework synthesis based on the Non-adoption, Abandonment, Scale-up, Spread and Sustainability framework (Figure 3).[33] These theoretical and empirical foundations will ensure data are structured in a way that accounts for the policy, organisational and practice level factors that influence implementation.[34] This will help to direct on going data collection, but also to rigorously curate data in preparation for thematic analysis.[35] Selection of a theoretical approach for data analysis will be informed by the data and systematic libraries of theoretical approaches used in clinical AI qualitative research and implementation research generally.[36, 37] COREQ reporting guidelines will be used.[38]

Patient and public involvement statement

The design of this protocol was informed by patient and carer members of the UK Macular Society and a National Institute for Health and Social Care Research (NIHR) funded lay consumer panel. These groups highlighted opportunities to improve current care by reducing travel requirements for a population commonly affected by other health and social challenges and reducing the congestion of clinics and the time that patients spend there. The project continues to benefit from a study advisory group including a nAMD patient and carer who use the NuTH service and a reference group including four members of the public from across the UK. These individuals have helped design the topic guide and will continue to advise on sampling, data collection, analysis and interpretation.

For peer review only - http://bmjopen.bmj.com/site/about/guidelines.xhtml

Ethics and dissemination

The study has received NHS Research Ethics Committee and UK Health Research Authority approvals (21/NW/0138). Though direct public access to data from the study is not supported, this ethics approval includes a means of sharing anonymised data following requests to the corresponding author and an application process. Dissemination is planned to clinical, academic and commercial stakeholders through peer reviewed conference presentation and open access journal publication. Public dissemination is planned through NIHR and macular society communication channels and a NIHR funded event in Newcastle upon Tyne for patients and their carers.

For peer review only

For peer review only - http://bmjopen.bmj.com/site/about/guidelines.xhtml

Author contributions

HDJH led funding application and drafted the manuscript. All authors contributed to the study design. DT, KB, SJT, PAK and GM reviewed and revised the manuscript.

Funding statement

This study was part of a proposal funded by a National Institute for Health Research (NIHR) doctoral fellowship (NIHR301467). The funder had no role in study design, data collection, data analysis, data interpretation or manuscript writing.

Competing interests statement

Google Health™ and Deepmind ™ are supporting this project through access to their analytical software at no charge.

Wordcount

3122/4000

For peer review only

Legends to figures

Figure 1. Process map of current 'one-stop' macular clinic visits for a patient. Some steps have the potential to be substituted by AI-enabled treatment decisions based on OCT imaging (thick black line), some could be relocated to primary care optometry (thin black line) and some could also be decentralised using community outreach treatment services (dashed black line).

Figure 2. Forest plot template for the relative negative predictive value (NPV) of artificial intelligence (AI)-enabled reports of neovascular age-related macular degeneration (nAMD) disease activity versus judgements from consultant-led-care relative to an enhanced reference standard from Moorfields Reading Centre. The non-inferiority and superiority margins are marked by dashed vertical lines on a logarithmic scale at 0.90 and 1.11 respectively. Potential outcomes for the non-inferiority test include scenario (a) AI-enabled reports are inferior to judgements from consultant-led-care; (b) Non-inferiority of AI-enabled reports to judgements from consultant-led-care is not demonstrated; (c) AI-enabled reports are non-inferior to judgements from consultant-led-care; (d) AI-enabled reports are non-inferior to judgements from consultant-led-care but not superior; (e) AI-enabled reports are superior to judgements from consultant-led-care.

Figure 3. Schematic of the Non-adoption, Abandonment, Scale-up, Spread and Sustainability (NASSS) framework. [33]

For peer review only - http://bmjopen.bmj.com/site/about/guidelines.xhtml

References

1. The Royal College of Ophthalmologists, Work Force Census 2018.

2. NHS Digital, Hospital Outpatient Activity. 2021.

3. Li, J.Q., et al., Prevalence and incidence of age-related macular degeneration in Europe: a systematic review and meta-analysis. British Journal of Ophthalmology, 2020. 104(8): p. 1077.

4. The Royal National Institute for the Blind, Sight loss data tool. 2021.

5. Hogg, J., et al., The prevalence and impact of treatment delays in exudative age-related macular degeneration. Investigative Ophthalmology & Visual Science, 2021. 62(8): p. 2941-2941.

6. Talks, J.S., et al., Appropriateness of quality standards for meaningful intercentre comparisons of aflibercept service provision for neovascular age-related macular degeneration. Eye (Lond), 2017. 31(11): p. 1613-1620.

7. Arruabarrena, C., et al., Impact on visual acuity in neovascular age related macular degeneration (Namd) in europe due to covid-19 pandemic lockdown. Journal of Clinical Medicine, 2021. 10(15).

8. Muehlematter, U.J., et al., Approval of artificial intelligence and machine learning-based medical devices in the USA and Europe (2015–20): a comparative analysis. The Lancet Digital Health, 2021. 3(3): p. e195-e203.

9. World Health Organisation, Generating evidence for artificial intelligence-based medical devices: A framework for training validation and evaluation. 2021.

For peer review only

10. Guo, Y., et al., Artificial Intelligence in Health Care: Bibliometric Analysis. J Med Internet Res, 2020. 22(7): p. e18228.

11. Liu, X., et al., A comparison of deep learning performance against health-care professionals in detecting diseases from medical imaging: a systematic review and meta-analysis. The Lancet Digital Health, 2019. 1(6): p. e271-e297.

12. De Fauw, J., et al., Clinically applicable deep learning for diagnosis and referral in retinal disease. Nature Medicine, 2018. 24(9): p. 1342-1350.

13. Han J.E.D., et al., Teleophthalmology-enabled and artificial intelligence-ready referral pathway for community optometry referrals of retinal disease (HERMES): a Cluster Randomised Superiority Trial with a linked Diagnostic Accuracy Study—HERMES study report 1—study protocol; *BMJ Open* 2022;12:e055845.

14. Blandford, A., et al., Protocol for a qualitative study to explore acceptability, barriers and facilitators of the implementation of new teleophthalmology technologies between community optometry practices and hospital eye services. BMJ Open, 2022. 12(7): p. e060810.

15. National Institute for Health and Care Excellence, Age-related macular degeneration; NICE guideline 82. 2018.

16. Ross, A.H., et al., Recommendations by a UK expert panel on an aflibercept treat-and-extend pathway for the treatment of neovascular age-related macular degeneration. Eye, 2020. 34(10): p. 1825-1834.

17. Bolme, S., et al., Task shifting of intraocular injections from physicians to nurses: a randomized single-masked noninferiority study. Acta Ophthalmol, 2020. 98(2): p. 139-144.

For peer review only - http://bmjopen.bmj.com/site/about/guidelines.xhtml

18. Hasan, H., S. Mamtora, and N. Shah, Setting up a successful nurse-led intravitreal injections service: Pearls from Swindon. British Journal of Nursing, 2020. 29(20): p. 1178-1185.

19. González-Gonzalo, C., et al., Trustworthy AI: Closing the gap between development and integration of AI systems in ophthalmic practice. Progress in Retinal and Eye Research, 2021: p. 101034.

20. von der Burchard, C., et al., Self-examination low-cost full-field OCT (SELFF-OCT) for patients with various macular diseases. Graefe's Archive for Clinical and Experimental Ophthalmology, 2021. 259(6): p. 1503-1511.

21. Amoaku, W., et al., Providing a safe and effective intravitreal treatment service: strategies for service delivery. Clinical Ophthalmology, 2020. 14: p. 1315-1328.

22. Skivington, K., et al., A new framework for developing and evaluating complex interventions: update of Medical Research Council guidance. BMJ, 2021. 374: p. n2061.

23. Learoyd, A.E., et al., FENETRE study: quality-assured follow-up of quiescent neovascular age-related macular degeneration by non-medical practitioners: study protocol and statistical analysis plan for a randomised controlled trial. BMJ Open, 2021. 11(5): p. e049411.

24. Moraes, G., et al., Quantitative Analysis of OCT for Neovascular Age-Related Macular Degeneration Using Deep Learning. Ophthalmology, 2021. 128(5): p. 693-705.

25. Moskowitz, C.S. and M.S. Pepe, Comparing the predictive values of diagnostic tests: sample size and analysis for paired study designs. Clinical Trials, 2006. 3(3): p. 272-279.

26. Teare, M.D., et al., Sample size requirements to estimate key design parameters from external pilot randomised controlled trials: a simulation study. Trials, 2014. 15: p. 264.

For peer review only

For peer review only - http://bmjopen.bmj.com/site/about/guidelines.xhtml

27. Reeves, B.C., et al., Effectiveness of Community versus Hospital Eye Service follow-up for patients with neovascular age-related macular degeneration with quiescent disease (ECHoES): a virtual non-inferiority trial. BMJ Open, 2016. 6(7): p. e010685.

28. Bossuyt, P.M., et al., STARD 2015: an updated list of essential items for reporting diagnostic accuracy studies. Bmj, 2015. 351: p. h5527.

29. Sounderajah, V., et al., Developing a reporting guideline for artificial intelligence-centred diagnostic test accuracy studies: the STARD-AI protocol. BMJ Open, 2021. 11(6): p. e047709.

30. Al-Zubaidy, M., et al., Stakeholder Perspectives on Clinical Decision Support Tools to Inform Clinical Artificial Intelligence Implementation: Protocol for a Framework Synthesis for Qualitative Evidence. JMIR Res Protoc, 2022. 11(4): p. e33145.

31. Richards, H. and C. Emslie, The 'doctor' or the 'girl from the University'? Considering the influence of professional roles on qualitative interviewing. Family Practice, 2000. 17(1): p. 71-75.

32. Ortlipp, M., Keeping and using reflective journals in the qualitative research process. The Qualitative Report, 2008. 13(4): p. 695-705.

33. Greenhalgh, T., et al., Beyond Adoption: A New Framework for Theorizing and Evaluating Nonadoption, Abandonment, and Challenges to the Scale-Up, Spread, and Sustainability of Health and Care Technologies. J Med Internet Res, 2017. 19(11): p. e367.

34. Maniatopoulos, G., et al., Moving beyond local practice: Reconfiguring the adoption of a breast cancer diagnostic technology. Social Science & Medicine, 2015. 131: p. 98-106.

35. Braun, V., et al., Using thematic analysis in psychology. Qualitative Research in Psychology, 2006. 3(2): p. 77-101.

For peer review only

For peer review only - http://bmjopen.bmj.com/site/about/guidelines.xhtml

36. Rabin, B., et al. Dissemination and Implementation Models in Health Research and Practice 2021; Available from: https://dissemination-implementation.org.

37. Hogg, H.D.J., et al., Implementing Implementation Science; A Bibliometric Study of Qualitative Research in Clinical Artificial Intelligence. Research Square, 2022.

38. Tong, A., P. Sainsbury, and J. Craig, Consolidated criteria for reporting qualitative research (COREQ): a 32-item checklist for interviews and focus groups. Int J Qual Health Care, 2007. 19(6): p. 349-57.

For peer review only

For peer review only - http://bmjopen.bmj.com/site/about/guidelines.xhtml

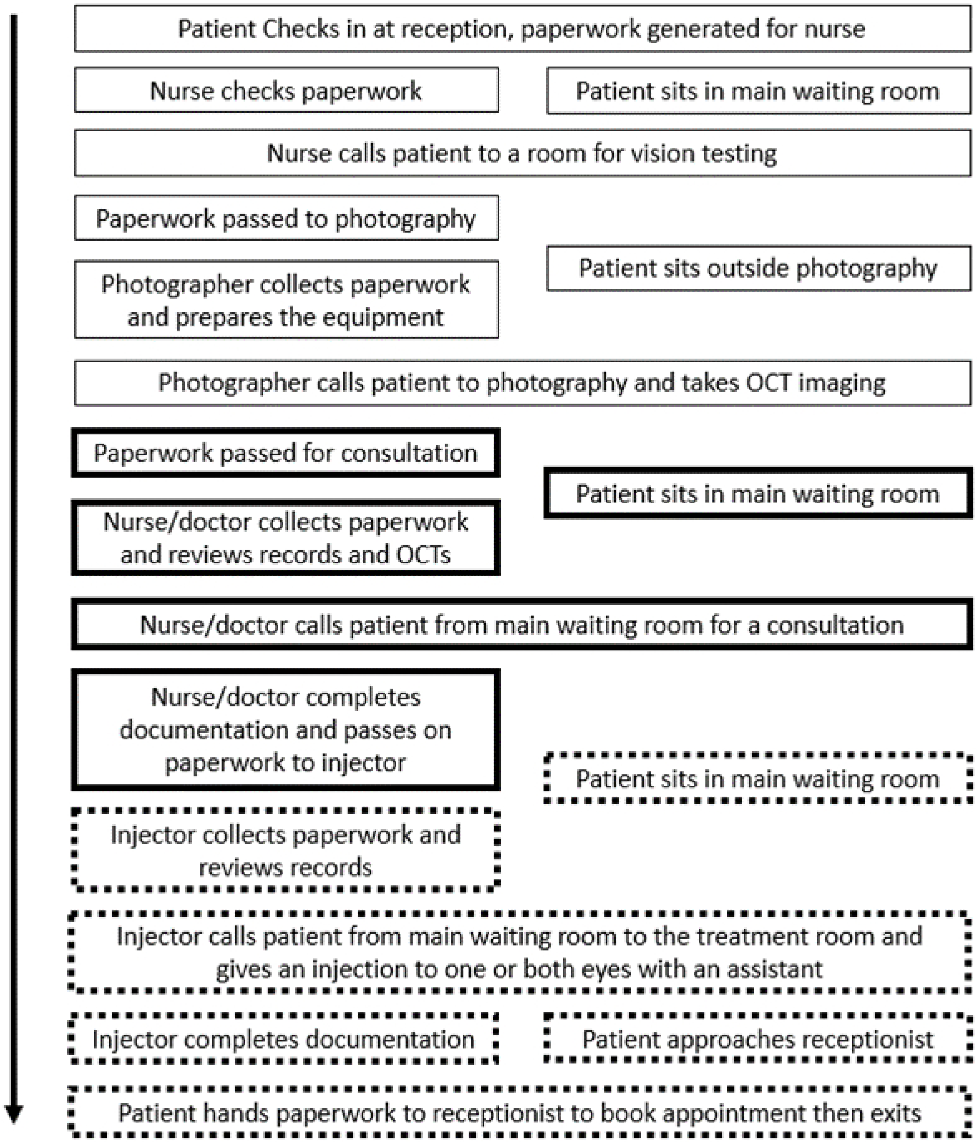

Process map of current 'one-stop' macular clinic visits for a patient. Some steps have the potential to be substituted by AI-enabled treatment decisions based on OCT imaging (thick black line), some could be relocated to primary care optometry (thin black line) and some could also be decentralised using community outreach treatment services (dashed black line).

402x467mm (38 x 38 DPI)

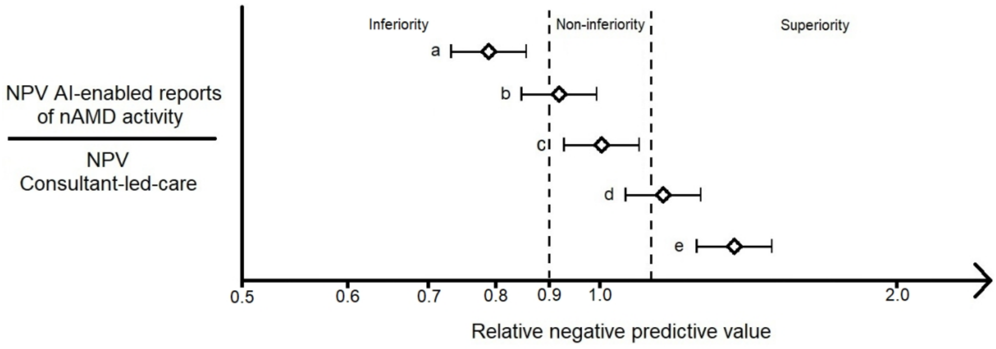

Forest plot template for the relative negative predictive value (NPV) of artificial intelligence (AI)-enabled reports of neovascular age-related macular degeneration (nAMD) disease activity versus judgements from consultant-led-care relative to an enhanced reference standard from Moorfields Reading Centre. The non-inferiority and superiority margins are marked by dashed vertical lines on a logarithmic scale at 0.90 and 1.11 respectively. Potential outcomes for the non-inferiority test include scenario (a) AI-enabled reports are inferior to judgements from consultant-led-care; (b) Non-inferiority of AI-enabled reports to judgements from consultant-led-care is not demonstrated; (c) AI-enabled reports are non-inferior to judgements from consultant-led-care; (d) AI-enabled reports are non-inferior to judgements from consultant-led-care but not superior; (e) AI-enabled reports are superior to judgements from consultant-led-care.

607x210mm (57 x 57 DPI)

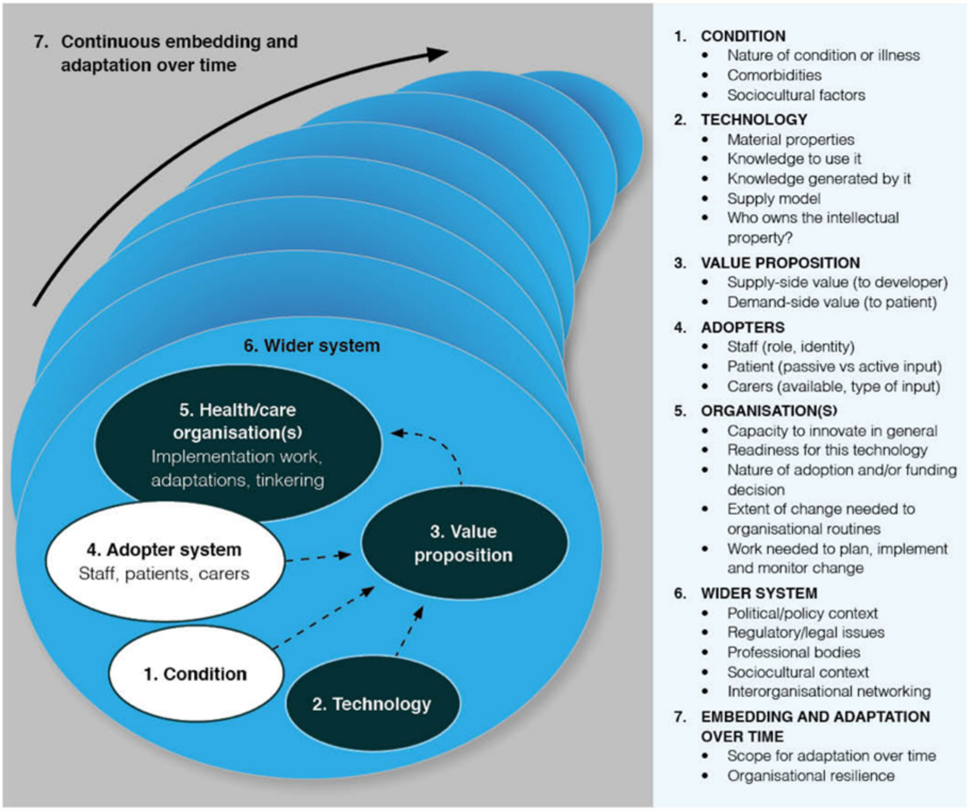

Schematic of the Non-adoption, Abandonment, Scale-up, Spread and Sustainability (NASSS) framework. [33]

404x334mm (59 x 59 DPI)

Systematic sampling strategy

Step	Question	Action if... Yes	Action if... No
1	Does this eye have no retinal diagnosis beside AMD or is it enrolled in a study?	Go to step 2	Reject this patient
2	Is this visit more than 10 weeks after the eye's first IVI?	Go to step 3	Switch to the next visit, go to step 2
3	Does this visit involve anti-VEGF treatment for nAMD?	Go to step 4	Switch to the next visit, go to step 2
4	Is this visit conducted under the loading or TEX protocols?	Go to step 5	Switch to the next visit, go to step 2
5	Are the VAs of interest free from the influence of other interventions?	Go to step 6	Switch to the next visit, go to step 2
6	Does this visit have an accompanying consultation recorded?	Go to step 7	Switch to the next visit, go to step 2
7	Is the treatment interval stated?	Go to step 8	Switch to the next visit, go to step 2
8	Is there a VA available for this visit and the prior?	Go to step 9	Switch to the next visit, go to step 2
9	Are there co-located 25 slice fovea-centred OCTs available for this visit and the prior?	Collect data	Switch to the next visit, go to step 2

AMD = Age-related macular degeneration, IVI = Intravitreal Injection, VEGF = Vascular Endothelial Growth Factor, nAMD = neovascular Age-related macular degeneration, TEX = Treat and Extend, VA = Visual Acuity, OCT = Optical Coherence Tomography

For peer review only

BMJ Open

Safety and efficacy of an artificial intelligence-enabled decision tool for treatment decisions in neovascular age-related macular degeneration and an exploration of clinical pathway integration and implementation: protocol for a multi-methods validation study

Journal:	*BMJ Open*
Manuscript ID	bmjopen-2022-069443.R1
Article Type:	Protocol
Date Submitted by the Author:	21-Dec-2022
Complete List of Authors:	Hogg, Henry; University of Newcastle upon Tyne; Newcastle Upon Tyne Hospitals NHS Foundation Trust<br>Brittain, Katie; University of Newcastle upon Tyne<br>Teare, Dawn; University of Newcastle upon Tyne<br>Talks, S.; Newcastle Upon Tyne Hospitals NHS Foundation Trust<br>Balaskas, Konstantinos; Moorfields Eye Hospital NHS Foundation Trust; University College London Institute of Ophthalmology<br>Keane, Pearse ; University College London Institute of Ophthalmology; Moorfields Eye Hospital City Road Campus<br>Maniatopoulos, Gregory; Northumbria University; Newcastle University Faculty of Medical Sciences
**Primary Subject Heading**:	Ophthalmology
Secondary Subject Heading:	Health informatics, Diagnostics, Evidence based practice, Medical management, Qualitative research
Keywords:	QUALITATIVE RESEARCH, Medical retina < OPHTHALMOLOGY, Organisation of health services < HEALTH SERVICES ADMINISTRATION & MANAGEMENT, Health informatics < BIOTECHNOLOGY & BIOINFORMATICS, STATISTICS & RESEARCH METHODS

SCHOLARONE™
Manuscripts

Safety and efficacy of an artificial intelligence-enabled decision tool for treatment decisions in neovascular age-related macular degeneration and an exploration of clinical pathway integration and implementation: protocol for a multi-methods validation study

Short title: AI-enabled macular service validation

Authors: Dr H.D. Jeffry Hogg[1,2,3]

Prof K. Brittain[1]

Prof D. Teare[1]

Mr. S.J. Talks[2]

Mr. K. Balaskas[3,4]

Prof P.A. Keane[3,4]

Dr G. Maniatopoulos[5,1]

Institutions: 1. The University of Newcastle upon Tyne, Newcastle upon Tyne, Tyne and Wear, NE1 7RU, U.K.

2. The Newcastle upon Tyne Hospitals NHS Foundation Trust, Newcastle upon Tyne, Tyne and Wear, NE1 7RU, U.K.

For peer review only - http://bmjopen.bmj.com/site/about/guidelines.xhtml

3. Moorfields Eye Hospital NHS Foundation Trust, 162 City Road, London, EC1V 2PD, U.K.

4. University College London Institute of Ophthalmology, 11-43 Bath Street, London, EC1V 9EL, U.K.

5. Northumbria University, Sutherland Building, 2 Ellison Place, Newcastle upon Tyne, NE1 8ST, U.K.

Corresponding: Jeffry Hogg, Newcastle Eye Centre, Royal Victoria Infirmary, Queen Victoria Rd, Newcastle upon Tyne, Tyne and Wear, NE1 4LP, U.K. Tel: 07900603591, Fax: 01912826269, Email: Jeffry.hogg@ncl.ac.uk

Financial support: This work is funded entirely by a National Institute of Health Research Doctoral Fellowship award; NIHR301467

Conflicts of interest: The authors declare no competing interests.

For peer review only

Abstract

Introduction

Neovascular age-related macular degeneration (nAMD) management is one of the largest single-disease contributors to hospital outpatient appointments. Partial automation of nAMD treatment decisions could reduce demands on clinician time. Established artificial intelligence (AI)-enabled retinal imaging analysis tools, could be applied to this use-case, but are not yet validated for it. A primary qualitative investigation of stakeholder perceptions of such an AI-enabled decision tool is also absent. This multi-methods study aims to establish the safety and efficacy of an AI-enabled decision tool for nAMD treatment decisions and understand where on the clinical pathway it could sit and what factors are likely to influence its implementation.

Methods and analysis

Single-centre retrospective imaging and clinical data will be collected from nAMD clinic visits at an NHS teaching hospital ophthalmology service, including judgements of nAMD disease stability or activity made in real-world consultant-led-care. Dataset size will be set by a power calculation using the first 127 randomly sampled eligible clinic visits. An AI-enabled retinal segmentation tool and a rule-based decision tree will independently analyse imaging data to report nAMD stability or activity for each of these clinic visits. Independently, an external reading centre will receive both clinical and imaging data to generate an enhanced reference standard for each clinic visit. The non-inferiority of the relative negative predictive value of AI-enabled reports on disease activity relative to consultant-led-care judgements will then be tested. In parallel, approximately 40 semi-structured interviews will be conducted with key nAMD service stakeholders, including patients. Transcripts will be coded using a theoretical framework and thematic analysis will follow.

Ethics and dissemination

For peer review only - http://bmjopen.bmj.com/site/about/guidelines.xhtml

NHS Research Ethics Committee and UK Health Research Authority approvals are in place (21/NW/0138). Informed consent is planned for interview participants only. Written and oral dissemination is planned to public, clinical, academic and commercial stakeholders.

Keywords:

Age-related macular degeneration, non-inferiority study, AI validation, qualitative research, semi-structure interviews, implementation, fusion data

Strengths and limitations of this study

- This non-inferiority study examines an AI use-case supported by patients and the public, with scope for improving the efficiency with which limited human resources are applied across the NHS and elsewhere.
- This AI use-case has a high degree of explainability as it relies on reviewable tissue segmentation and a simple rule-based decision tree which mimic widely used treatment paradigms.
- The multi-methods approach allows insights beyond efficacy alone to consider the effectiveness, mechanisms and system impacts of this complex intervention.
- Due to the pre-clinical nature of this study, stakeholder perspectives concerning this AI-use case will be hypothetical rather than from direct experience.
- As the non-inferiority study uses retrospective data only, the results will not reflect the challenges of real-time AI deployment which will require onward study.

For peer review only - http://bmjopen.bmj.com/site/about/guidelines.xhtml

Introduction

A threat to the efficacy of UK National Health Service (NHS) ophthalmology services is posed by insufficient ophthalmologist availability, in the face of a growing clinical need.[1, 2] Ophthalmology continued to incur more clinic appointments than any other NHS specialty through the Covid-19 pandemic, with age related macular degeneration (AMD) as the most common cause for these appointments.[2] AMD is the leading cause for certification of visual impairment in the UK and affects just over a quarter of Europeans aged over 60 to some extent.[3, 4] Only the disease course of a severe form of AMD, complicated by choroidal neovascularisation (nAMD), can benefit from current treatment and is thought to affect 1.4% of Europeans aged over 60.[3] Real-world data from a NHS centre showed that even before the impact of the Covid-19 pandemic, delay in delivering planned treatments for nAMD was common and associated with sight loss for the patients concerned.[5] This same NHS centre had recently displayed better nAMD visual outcomes than a mean of 12 large UK centres, suggesting the observed treatment delays are likely to be representative of the whole nation.[6] Since these observations, the Covid-19 pandemic has introduced additional nAMD treatment delays and avoidable sight loss, further developing the urgent need to augment clinical capacity for nAMD treatment.[7]

Clinical artificial intelligence (AI) is an increasing target of investment from government, industry and academia, often with the hope of increasing service capacity independent of the limits posed by clinician availability.[8-10] Ophthalmology is one of the most heavily researched specialties in AI medical image analysis with the non-contact infra-red based imaging modality, optical coherence tomography (OCT), accounting for much of this.[11] Retinal OCT scans form the substrate for several clinical AI tools, which anatomically segment the images to support various clinical decisions including triage and diagnosis.[12] The impact of these clinical AI functions on the efficacy and efficiency of primary to

For peer review only - http://bmjopen.bmj.com/site/about/guidelines.xhtml

secondary care referrals in ophthalmology are the subject of on-going prospective clinical studies.[13, 14] However, an even greater cumulative demand on ophthalmologists' time is making decisions about the treatment of nAMD within secondary care. Treatment takes the form of intravitreal injections (IVIs) of anti-vascular endothelial growth factor (anti-VEGF) agents which are recommended for variable periods of time and at varying intervals depending on changes in the retina observed through OCT.[15, 16] Whilst the decisions about treatments often involve ophthalmologists, the injections themselves are increasingly performed independently by specialised nurse practitioners and allied health professionals.[17, 18] Given the highly protocolised, OCT-dependent, nAMD treatment pathway and pre-existent clinical AI capable of independently quantifying the OCT features which inform that treatment, there is a clear opportunity for AI-enabled nAMD services to expand capacity and reduce avoidable real-world sight loss.[12, 16]

Despite this tension for change, clinical AI is yet to be implemented within routine NHS ophthalmic practice. This is at least partly due to the lack of evidence to forecast the effectiveness of clinical AI in the real-world setting and how it will interact with the various stakeholders and contexts which must host the technology.[19] Alongside other innovations such as portable OCT technology and community outreach treatment services, AI may enable even greater changes to the way in which macular services are delivered (Figure 1).[20, 21] The complexity derived from these interdependent technologies, contexts and stakeholders must be better understood if implementation is to optimise patient benefit from AI-enabled nAMD services. Given the years for which quantitative evidence of efficacy has existed for many clinical AI tools without subsequent real-world adoption, it may be that qualitative explorations of the complex implementation context are in fact the more urgent requirement.[11,22,23]

For peer review only - http://bmjopen.bmj.com/site/about/guidelines.xhtml

For peer review only

This single-centre multi-methods study aims to generate the necessary evidence by an external validation of the technology with real-world data and exploring the interdependent factors which will influence the implementation of AI-enabled nAMD services. In doing so, we aim to move beyond efficacy to consider intervention effectiveness and construct a theoretical basis to guide future implementation strategies.[22]

A quantitative study will be performed to test the non-inferiority of AI-enabled reports of nAMD disease activity against judgements made in consultant-led-care. In addition, a qualitative study based on semi-structured interviews with key stakeholders and patients will be conducted to explore where the clinical AI could sit within the nAMD care pathway and the factors likely to influence its implementation.

For peer review only

Methods and analysis

Quantitative methods

Sampling method

The electronic medical record (EMR) at Newcastle upon Tyne Hospitals NHS Foundation Trust (NuTH) will be searched to identify clinic visits where individuals received anti-VEGF IVI to treat nAMD. Patients at NuTH are treated under three different regimens dependent upon the length of their diagnosis and joint decisions between clinicians and patients: loading, treat-and-extend and pro-re-nata (Panel 1). The treatment intervals most commonly vary between 4 and 16 weeks. During the period through which data will be collected aflibercept and ranibizumab were the anti-VEGF treatments in use for nAMD.

For peer review only - http://bmjopen.bmj.com/site/about/guidelines.xhtml

Loading protocol: Starts at diagnosis of nAMD and consists of three anti-VEGF IVIs at four-week intervals (visits two and three do not include consultation or imaging), followed by IVIs at eight-week intervals, or less if signs of disease activity persist, for the remainder of the year.

Treat-and-extend (TEX) protocol: Starts one year after treatment initiation and dictates that the interval of anti-VEGF IVIs is increased in two-week increments until evidence of disease activity is noted at which point the treatment interval is reduced. If extension beyond a certain interval is noted to result in observable disease activity a (unspecified) number of times, then that interval ceases to be modified.

Pro-re-nata (PRN) protocol: Initiated as a joint decision for patients who appear to have little or no disease activity having been on one of the other two protocols. Here it is not assumed that an IVI will be given at each review, but only if evidence of disease activity is noted. The observation of returning disease activity may also lead to the return to one of the other two protocols.

Panel 1. Summary of neovascular age-related macular degeneration (nAMD) treatment at Newcastle upon Tyne Hospitals NHS Foundation Trust. IVI=Intravitreal Injection, VEGF = Vascular Endothelial Growth Factor

Eligible visits from this dataset of 70,884 will be randomly selected for manual review of patient EMR files to screen against eligibility criteria (Panel 2). These criteria exclude clinical visits that took place before current treatment and OCT imaging protocols were established or after treatment decisions may have been influenced by the Covid-19 pandemic. They also exclude visits conducted under the PRN treatment protocol as all visits on this protocol meeting the inclusion criterion for a same day anti-VEGF IVI must have been judged by the clinician to show disease activity and will therefore not be representative of

For peer review only - http://bmjopen.bmj.com/site/about/guidelines.xhtml

PRN clinic visits generally. To maximise their relevance to the research question alongside feasibility and rigour within a complex real-world dataset, the eligibility criteria and systematic screening approach through which they will be applied was iteratively designed and trialled by authors with clinical, operational and statistical expertise inside and outside of NuTH (HDJH, SJT, PAK, KBa and DT) (Supplementary file 1). Having reached consensus on the eligibility criteria and the screening approach, a single researcher with 9 years of clinical experience at NuTH will perform data collection (HDJH), to support consistency of their application and ensure fluency with local clinical and digital practices.

For peer review only

For peer review only - http://bmjopen.bmj.com/site/about/guidelines.xhtml

Inclusion criteria

- **Eye diagnosed with nAMD**
- **One or more prior anti-VEGF IVI at NuTH**
- Co-located 25 slice, fovea centred OCT imaging available for both the included and prior visits
- Clinic visit note states intended IVI interval
- Clinic visit included same-day IVI

Exclusion criteria

- **Retinal diagnosis other than nAMD in included eye**
- Visit before 2016
- Visit during or after March 2020
- Visits conducted under the pro-re-nata treatment protocol

Panel 2. Eligibility criteria for patients (in bold) and clinic visits. nAMD = Neovascular age-related macular degeneration, IVI = Intravitreal injection, NuTH = Newcastle upon Tyne Hospitals, OCT = Optical coherence tomography

Data collection and processing

For each included clinic visit the following data will be recorded to characterise the dataset:

- Anonym for the individual
- Eye laterality
- Gender
- Self-reported ethnicity
- Home address postal code stem
- Individual's age at that visit

For peer review only - http://bmjopen.bmj.com/site/about/guidelines.xhtml

The following will be recorded to send anonymised to Moorfields Reading Centre to generate a report of disease stability or activity to act as an enhanced reference standard for each visit. This information will also facilitate more meaningful post-hoc error analysis to explore the mechanisms of failure which the AI-enabled tool may exhibit. The findings from these analyses are a secondary outcome of the study and will help to delineate any groups of cases for which the tool's performance needs to be monitored and improved in further work, or for which only clinician judgements should be applied. This list was developed through additions to a proforma from a recent exemplar protocol:[24]

- OCT and visual acuity (VA) for that visit and the prior
- VA for the fellow eye at that visit
- Time since first nAMD treatment at that visit
- Total IVIs for nAMD in that eye up until that visit
- Observed interval since that eye's last IVI
- Presence or absence of evolving macular haemorrhage being recorded
- Time since increasing disease activity was last observed
- Treatment interval associated with that observation

The following data will also be recorded from each consultant-led-care visit to assess the primary and secondary outcomes and will not be sent to Moorfields Reading Centre:

- Judgement of disease activity or stability
- Planned interval to next IVI
- Professional group conducting the consultation
- Treatment protocol the visit was conducted under (Panel 1)

Separately, the present and prior pairs of OCT images relating to the same clinic visits will be transferred to Moorfields Eye Hospital NHS Foundation Trust for AI-enabled retinal

For peer review only - http://bmjopen.bmj.com/site/about/guidelines.xhtml

segmentation.[12] The differences in retinal tissue volumes will be used in a rule-based decision tree to produce an AI-enabled binary report of disease activity or stability for each included visit.

AI-enabled decision tool

The intervention to be tested on retrospective OCT imaging data is a deep learning tool with a U-net architecture with previously published details of training and validation.[12] It can produce volume quantification for the neurosensory retina, retinal pigment epithelium, fibrovascular pigment epithelium detachment, drusenoid pigment epithelium detachment, subretinal hyper-reflective material, subretinal fluid, intraretinal fluid, posterior hyaloid, epiretinal membrane and serous pigment epithelium detachment. An initial decision tree will report disease activity when the volumes of neurosensory retina, subretinal fluid or intraretinal fluid increase >5% between the current and prior visit OCTs.[25] This decision logic is based on a recent consensus from UK medical retina experts on treat-and-extend protocols for nAMD.[16] The exact tissue group contributors and decision thresholds for inter-visit changes in each of these tissue groups will be iterated upon using an embedded pilot dataset described further below. This binary output was preferred over a scalar recommendation of treatment interval to preserve the tools' value across different treatment protocols (Panel 1). The choice of a binary output will also help the generalisability of results, as the translation of OCT findings into treatment decisions varies internationally.

Outcomes measures

Over-treatment marginally increases the cumulative risk of IVI complications and the cost to the provider, but justifiably the main concern of patients and carers consulted in designing this study was sight loss through under-treatment.[5] Consequently, the probability of AI-enabled reports of disease stability being correct relative to judgements made in real-world consultant-led care has been taken as the most clinically relevant measure of diagnostic

For peer review only - http://bmjopen.bmj.com/site/about/guidelines.xhtml

accuracy. This has led to a non-inferiority design with the relative negative predictive value (NPV) as the primary outcome.

Secondary outcomes will be:

- A comparison of other standard diagnostic accuracy metrics between AI-enabled reports of disease activity and judgements from consultant-led-care accompanied by confusion matrices (Table 1)
- A comparison of the diagnostic accuracy of the five healthcare professional groups conducting consultations in consultant-led-care (nurses, optometrists, ophthalmology specialty trainees, medical retina sub-specialty fellows, medical retina sub-specialty consultants)
- A comparison of the treatment intervals recommended in real-world consultant-led care and the treatment intervals that would be derived from AI-enabled reports of disease activity given the treatment protocol
- A case-by-case exploration of false-positive and false-negative reports of disease activity from the AI-enabled decision tool and consultant-led care

Justification of study design and sample size

An enhanced reference standard is required to facilitate comparison between a potential AI-enabled nAMD service and the real-world gold standard, consultant-led-care. Moorfields Reading Centre has an international reputation and track record in meeting this need for prior studies and will receive imaging and clinical data for each case in the present study to generate an enhanced reference standard.[12, 13, 24] The binary decision under examination for each included visit is whether the data suggest disease stability or activity. This simplification of the scalar number of weeks between treatments or the three-option decision regarding treatment interval maintenance, extension or reduction is more broadly relevant to

For peer review only - http://bmjopen.bmj.com/site/about/guidelines.xhtml

real-world practice where clinician and patient preferences about how treatment intervals should be altered on account of disease activity vary (Panel 1).[16] It also lowers the risk of inappropriately labelling a decision made in consultant-led-care with subtle influences from patient or clinician preference as 'incorrect'. Whether binary judgements of disease activity (positives) or stability (negatives) from consultant-led-care and the AI-enabled decision tool are labelled as true or false will be decided by the independent judgement of the Moorfields Reading Centre (Table 1).

Table 1. Template confusion matrix showing the different possible classification of Artificial Intelligence (AI)-enabled reports of disease activity and judgements from consultant-led-care (CLC) for each eligible case.

	Moorfields Reading Centre identifies disease stability (negative)	Moorfields Reading Centre identifies disease activity (positive)
AI identifies disease stability (negative)	AI True negative	AI False negative
AI identifies disease activity (positive)	AI False positive	AI True positive
CLC identifies disease stability (negative)	CLC True negative	CLC False negative
CLC identifies disease activity (positive)	CLC False positive	CLC True positive

Treatment decision data to support power calculations are unavailable for this novel use case. Considering a different use case, the deep learning model to be applied within the proposed AI-enabled decision tool has demonstrated equivalent or superior retinal diagnostic performance to consultant specialists. Relative to final real-world clinical diagnoses it produced an area under the ROC curve of 99% for retinal diseases including nAMD.[12] This performance was dependent on the same intermediate anatomical segmentation step that will form the basis for the AI-enabled decision tool for nAMD treatment proposed here. This has supported the feasibility of the current proposal but does not provide the level of certainty required to perform a robust power calculation, which requires sufficiently accurate estimates of the NPV of both judgements from consultant-led-care and AI-enabled reports from paired

For peer review only - http://bmjopen.bmj.com/site/about/guidelines.xhtml

data.[26] Therefore, a pilot dataset will be collected and sent for independent processing by Moorfields Reading Centre and the AI-enabled decision tool to supply these estimates. Prior work has established that for binary outcomes such as the one under study, little improvement is seen in estimate precision or bias by increasing the size of the pilot dataset above 100.[27] A preliminary review of 100 NuTH consultant-led-care nAMD clinic visits under loading or TEX treatment protocols found that 79 reported disease stability, and would be classed as negative cases which could contribute to estimating NPV. Given that about 79% of eligible visits are classed as negative the randomly sampled pilot dataset required to accrue 100 negative cases is expected to be around 127 cases (100 / 0.79), but the consultant-led-care judgement for each case will be reviewed as it is curated to ensure the pilot dataset contains 100 negative cases. The estimated NPV of AI-enabled reports and judgements from consultant-led-care will be derived from this pilot dataset and inform a power calculation. From this, the number of additional eligible cases required to be collected and processed by Moorfields Reading Centre and the AI-enabled decision tool to test the non-inferiority of AI-enabled report NPV relative to judgements from consultant-led-care will be established.[26] This power calculation will include a significance level $\alpha = 0.05$, a power $\beta = 0.90$ and a relative non-inferiority margin of $\delta = 0.90$. Although this non-inferiority margin is relative, it will be similar and no larger than an absolute equivalent. This allowed the application of 10% non-inferiority margins applied in comparable studies.[24, 28] In evaluating this non-inferiority margin it is helpful to remember that the least desirable outcome of a false negative would yield a two or four week delay for the next planned treatment rather than treatment cessation and that 22% of patients are estimated to experience more than four weeks of delay to treatment in a year in current consultant-led care.[5] As such the null hypothesis will be rejected if the lower confidence limit for the relative NPV of AI-enabled reports is greater than 0.90.[26] This will be visually presented with 2-sided 95% confidence

For peer review only - http://bmjopen.bmj.com/site/about/guidelines.xhtml

interval to explore the possibility of superiority with a threshold γ of 1/ δ; $\gamma = 1.11$ (Figure 2). Whilst the dataset will offer other important exploratory insights, the potential impact of the primary outcome on the translation of this AI-enabled decision tool and threat to the study's feasibility from ambitions outside this scope, has prevented any plans to proactively power the sample size for secondary outcomes.

Data analysis

The relative NPV of AI-enabled reports / judgements from consultant-led-care will be calculated with 95% confidence intervals to see if the inferiority, non-inferiority or superiority of AI-enabled reports of disease activity can be established (Table 1). For secondary outcomes, diagnostic accuracy statistics for each group will be reported descriptively with 95% confidence intervals for each group, along with confusion matrices. Clinical and imaging data from cases of false positives and false negatives of the AI-enabled reports will be reviewed by clinical members of the team, supported by the AI development team where necessary, to try to understand the mechanisms and potential consequences of AI-enabled decision tool failures. STARD reporting guidelines will be used alongside the STARD-AI extension if available at the time of writing.[29, 30]

Qualitative methods

Sampling

A recent systematic review outlined the importance of all stakeholder perspectives in understanding the interdependent factors that influence clinical AI implementation.[31,32] Despite this, the qualitative literature regarding clinical AI is dominated by healthcare professionals' perspectives and has particularly limited representation from carers.[31] Consequently, the present study will aim to explore perspective from all stakeholders. This will include patients from the NuTH nAMD clinic, carers of patients from the same clinic, doctors, nurses, photographers and optometrists working in the clinics, primary care doctors

For peer review only - http://bmjopen.bmj.com/site/about/guidelines.xhtml

and optometrists, hospital managers, relevant industry and charity sector professionals, and care commissioners. An approximate target of 40 participants has been set, but the targeting and scale of recruitment will be refined by themes and stakeholders identified in the data.

Data collection

Topic guides have been informed by the aforementioned systematic review and input from clinicians, academics, patients and lay members of the study team (Supplementary file 2). In their initial form these topic guides focus on:

- Stakeholders' experiences of the present macula service
- Their perceptions of clinical AI generally and specifically in recommending treatment intervals for nAMD
- Where this AI-enabled decision tool should be placed within the healthcare pathway and which cases or settings may not be appropriate
- Factors likely to affect the implementation of this AI-enabled decision tool
- How relationships between stakeholders and with the AI itself may change or develop

The topic guides will be used flexibly to guide semi-structure interviews and continuously iterated as data are analysed in parallel. Depending upon participant preference interviews may be conducted face-to-face or remotely by telephone or teleconferencing platforms. Interviews will be recorded and transcribed for text-based coding and analysis. Interviews will be delivered by the same researcher (HDJH) throughout, who is an ophthalmologist with lived experience of working in NuTH nAMD clinics and prior training and experience in qualitative research methods. Although patients with prior clinical contact with the interviewer will be ineligible for recruitment and the interviewer will be presented as a researcher rather than a clinician, their status as a healthcare professional may emerge unintentionally and could influence data elicitation.[33] It is also true that some of the

For peer review only - http://bmjopen.bmj.com/site/about/guidelines.xhtml

healthcare professional interviewees will have worked with the interviewer as a colleague in the past which is likely to influence data. Reflective journaling will be used to transparently report and manage these influences on data collection, analysis and interpretation.[34] Data will also be reviewed throughout the collection period by the wider study team, including lay representatives, to help identify and manage biases in sampling, data collection, analysis and interpretation.

Data analysis

The same researcher (HDJH) will familiarise themselves with data as it is collected and iteratively place data into a-priori codes and categories derived from a qualitative framework synthesis based on the Non-adoption, Abandonment, Scale-up, Spread and Sustainability framework (Figure 3).[35] These theoretical and empirical foundations will ensure data are structured in a way that accounts for the policy, organisational and practice level factors that influence implementation.[36] This will help to direct on going data collection, but also to rigorously curate data in preparation for thematic analysis.[37] Selection of a theoretical approach for data analysis will be informed by the data and systematic libraries of theoretical approaches used in clinical AI qualitative research and implementation research generally.[38, 39] COREQ reporting guidelines will be used.[40]

Patient and public involvement statement

The design of this protocol was informed by patient and carer members of the UK Macular Society and a National Institute for Health and Social Care Research (NIHR) funded lay consumer panel. These groups highlighted opportunities to improve current care by reducing travel requirements for a population commonly affected by other health and social challenges and reducing the congestion of clinics and the time that patients spend there. The project

For peer review only - http://bmjopen.bmj.com/site/about/guidelines.xhtml

continues to benefit from a study advisory group including a nAMD patient and carer who use the NuTH service and a reference group including four members of the public from across the UK. These individuals have helped design the topic guide and will continue to advise on sampling, data collection, analysis and interpretation.

Ethics and dissemination

The study has received NHS Research Ethics Committee and UK Health Research Authority approvals (21/NW/0138). Informed consent is planned for interview participants only. Though direct public access to data from the study is not supported, this ethics approval includes a means of sharing anonymised data following requests to the corresponding author and an application process. Dissemination is planned to clinical, academic and commercial stakeholders through peer reviewed conference presentation and open access journal publication. Public dissemination is planned through NIHR and macular society communication channels and a NIHR funded event in Newcastle upon Tyne for patients and their carers.

For peer review only

For peer review only - http://bmjopen.bmj.com/site/about/guidelines.xhtml

Author contributions

HDJH led funding application and drafted the manuscript. All authors contributed to the study design. DT, KBr, KBa SJT, PAK and GM reviewed and revised the manuscript.

Funding statement

This study was part of a proposal funded by a National Institute for Health Research (NIHR) doctoral fellowship (NIHR301467). The funder had no role in study design, data collection, data analysis, data interpretation or manuscript writing.

Competing interests statement

The authors declare no competing interests.

Wordcount

3375/4000

For peer review only

Legends to figures

Figure 1. Process map of current 'one-stop' macular clinic visits for a patient. Some steps have the potential to be substituted by AI-enabled treatment decisions based on OCT imaging (thick black line), some could be relocated to primary care optometry (thin black line) and some could also be decentralised using community outreach treatment services (dashed black line).

Figure 2. Forest plot template for the relative negative predictive value (NPV) of artificial intelligence (AI)-enabled reports of neovascular age-related macular degeneration (nAMD) disease activity versus judgements from consultant-led-care relative to an enhanced reference standard from Moorfields Reading Centre. The non-inferiority and superiority margins are marked by dashed vertical lines on a logarithmic scale at 0.90 and 1.11 respectively. Potential outcomes for the non-inferiority test include scenario (a) AI-enabled reports are inferior to judgements from consultant-led-care; (b) Non-inferiority of AI-enabled reports to judgements from consultant-led-care is not demonstrated; (c) AI-enabled reports are non-inferior to judgements from consultant-led-care; (d) AI-enabled reports are non-inferior to judgements from consultant-led-care but not superior; (e) AI-enabled reports are superior to judgements from consultant-led-care.

Figure 3. Schematic of the Non-adoption, Abandonment, Scale-up, Spread and Sustainability (NASSS) framework. [35]

For peer review only

For peer review only - http://bmjopen.bmj.com/site/about/guidelines.xhtml

References

1. The Royal College of Ophthalmologists, Work Force Census 2018.

2. NHS Digital, Hospital Outpatient Activity. 2021.

3. Li, J.Q., et al., Prevalence and incidence of age-related macular degeneration in Europe: a systematic review and meta-analysis. British Journal of Ophthalmology, 2020. 104(8): p. 1077.

4. The Royal National Institute for the Blind, Sight loss data tool. 2021.

5. Hogg, J., et al., The prevalence and impact of treatment delays in exudative age-related macular degeneration. Investigative Ophthalmology & Visual Science, 2021. 62(8): p. 2941-2941.

6. Talks, J.S., et al., Appropriateness of quality standards for meaningful intercentre comparisons of aflibercept service provision for neovascular age-related macular degeneration. Eye (Lond), 2017. 31(11): p. 1613-1620.

7. Arruabarrena, C., et al., Impact on visual acuity in neovascular age related macular degeneration (Namd) in europe due to covid-19 pandemic lockdown. Journal of Clinical Medicine, 2021. 10(15).

8. Muehlematter, U.J., et al., Approval of artificial intelligence and machine learning-based medical devices in the USA and Europe (2015–20): a comparative analysis. The Lancet Digital Health, 2021. 3(3): p. e195-e203.

9. World Health Organisation, Generating evidence for artificial intelligence-based medical devices: A framework for training validation and evaluation. 2021.

For peer review only

For peer review only - http://bmjopen.bmj.com/site/about/guidelines.xhtml

10. Guo, Y., et al., Artificial Intelligence in Health Care: Bibliometric Analysis. J Med Internet Res, 2020. 22(7): p. e18228.

11. Liu, X., et al., A comparison of deep learning performance against health-care professionals in detecting diseases from medical imaging: a systematic review and meta-analysis. The Lancet Digital Health, 2019. 1(6): p. e271-e297.

12. De Fauw, J., et al., Clinically applicable deep learning for diagnosis and referral in retinal disease. Nature Medicine, 2018. 24(9): p. 1342-1350.

13. Han J.E.D., et al., Teleophthalmology-enabled and artificial intelligence-ready referral pathway for community optometry referrals of retinal disease (HERMES): a Cluster Randomised Superiority Trial with a linked Diagnostic Accuracy Study—HERMES study report 1—study protocol; *BMJ Open* 2022;12:e055845.

14. Blandford, A., et al., Protocol for a qualitative study to explore acceptability, barriers and facilitators of the implementation of new teleophthalmology technologies between community optometry practices and hospital eye services. BMJ Open, 2022. 12(7): p. e060810.

15. National Institute for Health and Care Excellence, Age-related macular degeneration; NICE guideline 82. 2018.

16. Ross, A.H., et al., Recommendations by a UK expert panel on an aflibercept treat-and-extend pathway for the treatment of neovascular age-related macular degeneration. Eye, 2020. 34(10): p. 1825-1834.

17. Bolme, S., et al., Task shifting of intraocular injections from physicians to nurses: a randomized single-masked noninferiority study. Acta Ophthalmol, 2020. 98(2): p. 139-144.

For peer review only - http://bmjopen.bmj.com/site/about/guidelines.xhtml

18. Hasan, H., S. Mamtora, and N. Shah, Setting up a successful nurse-led intravitreal injections service: Pearls from Swindon. British Journal of Nursing, 2020. 29(20): p. 1178-1185.

19. González-Gonzalo, C., et al., Trustworthy AI: Closing the gap between development and integration of AI systems in ophthalmic practice. Progress in Retinal and Eye Research, 2021: p. 101034.

20. von der Burchard, C., et al., Self-examination low-cost full-field OCT (SELFF-OCT) for patients with various macular diseases. Graefe's Archive for Clinical and Experimental Ophthalmology, 2021. 259(6): p. 1503-1511.

21. Amoaku, W., et al., Providing a safe and effective intravitreal treatment service: strategies for service delivery. Clinical Ophthalmology, 2020. 14: p. 1315-1328.

22. Skivington, K., et al., A new framework for developing and evaluating complex interventions: update of Medical Research Council guidance. BMJ, 2021. 374: p. n2061.

23. Maniatopoulos, G., Hunter, D. J., Erskine, J., & Hudson, B. (2020). Implementing the New Care Models in the NHS: Reconfiguring the Multilevel Nature of Context to Make It Happen. In *Transitions and Boundaries in the Coordination and Reform of Health Services* (pp. 3-27). Palgrave Macmillan, Cham.

24. Learoyd, A.E., et al., FENETRE study: quality-assured follow-up of quiescent neovascular age-related macular degeneration by non-medical practitioners: study protocol and statistical analysis plan for a randomised controlled trial. BMJ Open, 2021. 11(5): p. e049411.

25. Moraes, G., et al., Quantitative Analysis of OCT for Neovascular Age-Related Macular Degeneration Using Deep Learning. Ophthalmology, 2021. 128(5): p. 693-705.

For peer review only - http://bmjopen.bmj.com/site/about/guidelines.xhtml

26. Moskowitz, C.S. and M.S. Pepe, Comparing the predictive values of diagnostic tests: sample size and analysis for paired study designs. Clinical Trials, 2006. 3(3): p. 272-279.

27. Teare, M.D., et al., Sample size requirements to estimate key design parameters from external pilot randomised controlled trials: a simulation study. Trials, 2014. 15: p. 264.

28. Reeves, B.C., et al., Effectiveness of Community versus Hospital Eye Service follow-up for patients with neovascular age-related macular degeneration with quiescent disease (ECHoES): a virtual non-inferiority trial. BMJ Open, 2016. 6(7): p. e010685.

29. Bossuyt, P.M., et al., STARD 2015: an updated list of essential items for reporting diagnostic accuracy studies. Bmj, 2015. 351: p. h5527.

30. Sounderajah, V., et al., Developing a reporting guideline for artificial intelligence-centred diagnostic test accuracy studies: the STARD-AI protocol. BMJ Open, 2021. 11(6): p. e047709.

31. Al-Zubaidy, M., et al., Stakeholder Perspectives on Clinical Decision Support Tools to Inform Clinical Artificial Intelligence Implementation: Protocol for a Framework Synthesis for Qualitative Evidence. JMIR Res Protoc, 2022. 11(4): p. e33145.

32. Hogg HDJ, Al-Zubaidy M, Technology Enhanced Macular Services Study Reference Group et al. Multi-stakeholder perspectives of clinical artificial intelligence implementation: systematic review of qualitative evidence. J Med Internet Res (forthcoming). doi:10.2196/39742

33. Richards, H. and C. Emslie, The ‘doctor’ or the ‘girl from the University’? Considering the influence of professional roles on qualitative interviewing. Family Practice, 2000. 17(1): p. 71-75.

For peer review only - http://bmjopen.bmj.com/site/about/guidelines.xhtml

34. Ortlipp, M., Keeping and using reflective journals in the qualitative research process. The Qualitative Report, 2008. 13(4): p. 695-705.

35. Greenhalgh, T., et al., Beyond Adoption: A New Framework for Theorizing and Evaluating Nonadoption, Abandonment, and Challenges to the Scale-Up, Spread, and Sustainability of Health and Care Technologies. J Med Internet Res, 2017. 19(11): p. e367.

36. Maniatopoulos, G., et al., Moving beyond local practice: Reconfiguring the adoption of a breast cancer diagnostic technology. Social Science & Medicine, 2015. 131: p. 98-106.

37. Braun, V., et al., Using thematic analysis in psychology. Qualitative Research in Psychology, 2006. 3(2): p. 77-101.

38. Rabin, B., et al. Dissemination and Implementation Models in Health Research and Practice 2021; Available from: https://dissemination-implementation.org.

39. Hogg, H.D.J., et al., Implementing Implementation Science; A Bibliometric Study of Qualitative Research in Clinical Artificial Intelligence. Research Square, 2022.

40. Tong, A., P. Sainsbury, and J. Craig, Consolidated criteria for reporting qualitative research (COREQ): a 32-item checklist for interviews and focus groups. Int J Qual Health Care, 2007. 19(6): p. 349-57.

For peer review only

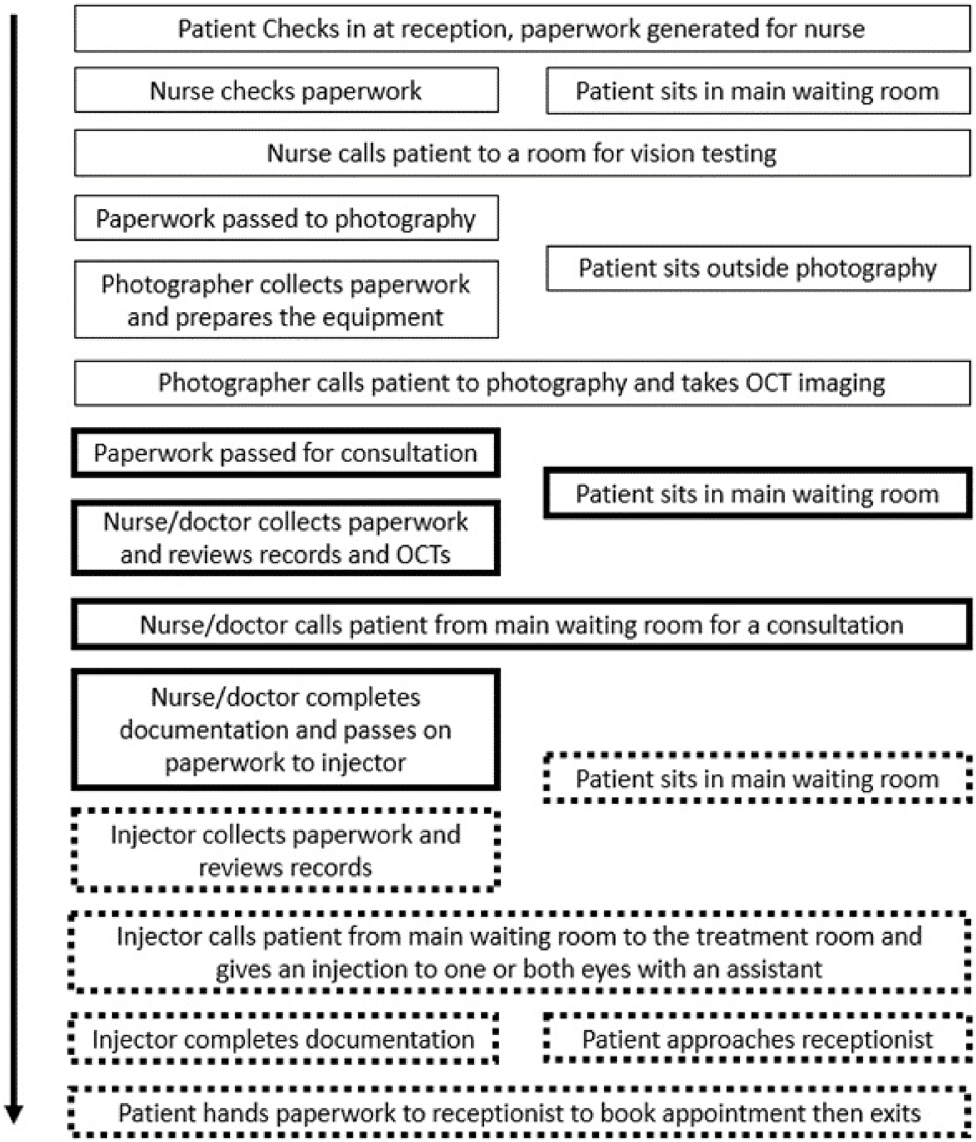

Process map of current 'one-stop' macular clinic visits for a patient. Some steps have the potential to be substituted by AI-enabled treatment decisions based on OCT imaging (thick black line), some could be relocated to primary care optometry (thin black line) and some could also be decentralised using community outreach treatment services (dashed black line).

106x123mm (144 x 144 DPI)

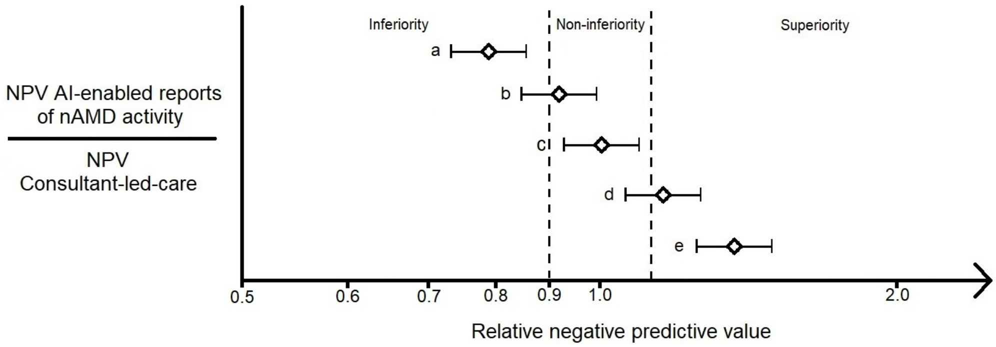

Forest plot template for the relative negative predictive value (NPV) of artificial intelligence (AI)-enabled reports of neovascular age-related macular degeneration (nAMD) disease activity versus judgements from consultant-led-care relative to an enhanced reference standard from Moorfields Reading Centre. The non-inferiority and superiority margins are marked by dashed vertical lines on a logarithmic scale at 0.90 and 1.11 respectively. Potential outcomes for the non-inferiority test include scenario (a) AI-enabled reports are inferior to judgements from consultant-led-care; (b) Non-inferiority of AI-enabled reports to judgements from consultant-led-care is not demonstrated; (c) AI-enabled reports are non-inferior to judgements from consultant-led-care; (d) AI-enabled reports are non-inferior to judgements from consultant-led-care but not superior; (e) AI-enabled reports are superior to judgements from consultant-led-care.

240x83mm (144 x 144 DPI)

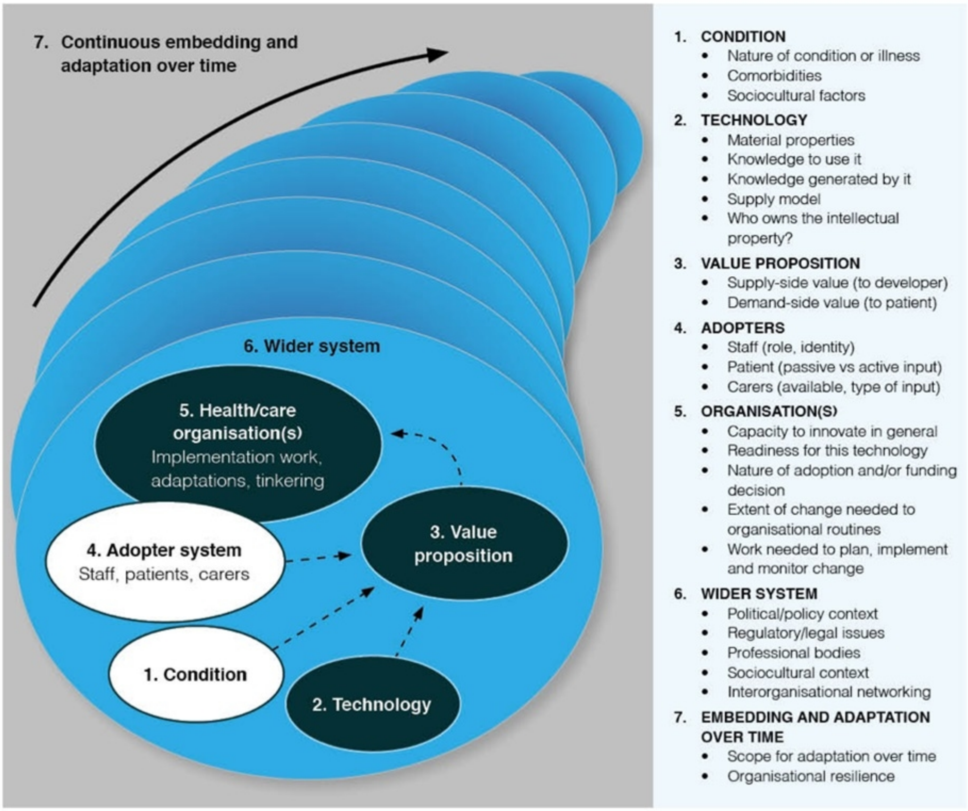

Figure 3. Schematic of the Non-adoption, Abandonment, Scale-up, Spread and Sustainability (NASSS) framework. [35]

165x137mm (144 x 144 DPI)

Systematic sampling strategy

Step	Question	Action if…	
		Yes	No
1	Does this eye have no retinal diagnosis beside AMD or is it enrolled in a study?	Go to step 2	Reject this patient
2	Is this visit more than 10 weeks after the eye's first IVI?	Go to step 3	Switch to the next visit, go to step 2
3	Does this visit involve anti-VEGF treatment for nAMD?	Go to step 4	Switch to the next visit, go to step 2
4	Is this visit conducted under the loading or TEX protocols?	Go to step 5	Switch to the next visit, go to step 2
5	Are the VAs of interest free from the influence of other interventions?	Go to step 6	Switch to the next visit, go to step 2
6	Does this visit have an accompanying consultation recorded?	Go to step 7	Switch to the next visit, go to step 2
7	Is the treatment interval stated?	Go to step 8	Switch to the next visit, go to step 2
8	Is there a VA available for this visit and the prior?	Go to step 9	Switch to the next visit, go to step 2
9	Are there co-located 25 slice fovea-centred OCTs available for this visit and the prior?	Collect data	Switch to the next visit, go to step 2

AMD = Age-related macular degeneration, IVI = Intravitreal Injection, VEGF = Vascular Endothelial Growth Factor, nAMD = neovascular Age-related macular degeneration, TEX = Treat and Extend, VA = Visual Acuity, OCT = Optical Coherence Tomography

For peer review only

Patient topic guide

Research question

What place do stakeholders in macular services think artificial intelligence ought to take in nAMD care pathways and what are the barriers and facilitators to its adoption there?

Topics and example questions

Current pathway

1. What parts of the current service do you like most?
2. What changes would you like to see in the current service?
3. In the current service you meet lots of team members for different parts of your care. Which bits of face-to-face or written contact do you find most valuable?
4. Have you had any 'injection only' or virtual clinic visits, where a consultation isn't part of the visit? If so, how do you find them?

Initial impressions of clinical AI

1. As you know, I'm interested in the idea of technology in healthcare. What are your first thoughts and feelings when I mention artificial intelligence in healthcare?
2. When I'm talking about AI here, I mean a type of technology designed to take in some information and make a decision about it on its own. Specifically, I'm talking about a kind of AI we have, which can look at your eye photos on its own and see when your eye next needs an injection without help from a nurse or doctor. What use could you imagine for AI like that?
3. What kind of down-sides or difficulties do you think there might be in using that kind of AI in the clinic?

Pathway placement

1. Who do you think the best person to be responsible for the AI would be?
2. What kind of interactions would you like to have with doctors if artificial intelligence is brought in?
3. Where would you like to have your eye photos taken?
4. Where would you like to have your injections given?

Relationships with the tool and others

1. Some people might feel a bit uncomfortable about letting AI take some of the responsibility for their treatment planning. What kind of things might help you trust AI like this?
2. With this kind of AI, you might be able to see how and why it makes its treatment decisions for you. How would you feel about that?
3. How might it change the way friends and family support you in managing your eye disease?
4. Who would you want to be able to access the AI's decision making?
5. How do you think bringing this AI into the service would affect your relationships with different members of the care team?

Closing

1. Thanks very much for so many helpful insights. Is there anything else we haven't talked about that seems important about using AI in macular degeneration clinics?
2. We're planning on talking to hospital doctors and nurses, opticians and managers but do you think there are other people's perspectives we should be hearing?

For peer review only

BMJ Open

Safety and efficacy of an artificial intelligence-enabled decision tool for treatment decisions in neovascular age-related macular degeneration and an exploration of clinical pathway integration and implementation: protocol for a multi-methods validation study

Journal:	*BMJ Open*
Manuscript ID	bmjopen-2022-069443.R2
Article Type:	Protocol
Date Submitted by the Author:	13-Jan-2023

Complete List of Authors:	Hogg, Henry; University of Newcastle upon Tyne; Newcastle Upon Tyne Hospitals NHS Foundation Trust<br>Brittain, Katie; University of Newcastle upon Tyne<br>Teare, Dawn; University of Newcastle upon Tyne<br>Talks, S.; Newcastle Upon Tyne Hospitals NHS Foundation Trust<br>Balaskas, Konstantinos; Moorfields Eye Hospital NHS Foundation Trust; University College London Institute of Ophthalmology<br>Keane, Pearse ; University College London Institute of Ophthalmology; Moorfields Eye Hospital City Road Campus<br>Maniatopoulos, Gregory; Northumbria University; Newcastle University Faculty of Medical Sciences

**Primary Subject Heading**:	Ophthalmology
Secondary Subject Heading:	Health informatics, Diagnostics, Evidence based practice, Medical management, Qualitative research
Keywords:	QUALITATIVE RESEARCH, Medical retina < OPHTHALMOLOGY, Organisation of health services < HEALTH SERVICES ADMINISTRATION & MANAGEMENT, Health informatics < BIOTECHNOLOGY & BIOINFORMATICS, STATISTICS & RESEARCH METHODS

SCHOLARONE™
Manuscripts

Safety and efficacy of an artificial intelligence-enabled decision tool for treatment decisions in neovascular age-related macular degeneration and an exploration of clinical pathway integration and implementation: protocol for a multi-methods validation study

Authors: Dr H.D. Jeffry Hogg[1,2,3]

Prof K. Brittain[1]

Prof D. Teare[1]

Mr. S.J. Talks[2]

Mr. K. Balaskas[3,4]

Prof P.A. Keane[3,4]

Dr G. Maniatopoulos[5,1]

Institutions: 1. The University of Newcastle upon Tyne, Newcastle upon Tyne, Tyne and Wear, NE1 7RU, U.K.

2. The Newcastle upon Tyne Hospitals NHS Foundation Trust, Newcastle upon Tyne, Tyne and Wear, NE1 7RU, U.K.

3. Moorfields Eye Hospital NHS Foundation Trust, 162 City Road, London, EC1V 2PD, U.K.

4. University College London Institute of Ophthalmology, 11-43 Bath Street, London, EC1V 9EL, U.K.

5. Northumbria University, Sutherland Building, 2 Ellison Place, Newcastle upon Tyne, NE1 8ST, U.K.

For peer review only

For peer review only - http://bmjopen.bmj.com/site/about/guidelines.xhtml

Correspondence to: Jeffry Hogg, Newcastle Eye Centre, Royal Victoria Infirmary, Queen Victoria Rd, Newcastle upon Tyne, Tyne and Wear, NE1 4LP, U.K.

Tel: 07900603591, Fax: 01912826269, Email: Jeffry.Hogg@ncl.ac.uk

For peer review only

For peer review only - http://bmjopen.bmj.com/site/about/guidelines.xhtml

Abstract

Introduction

Neovascular age-related macular degeneration (nAMD) management is one of the largest single-disease contributors to hospital outpatient appointments. Partial automation of nAMD treatment decisions could reduce demands on clinician time. Established artificial intelligence (AI)-enabled retinal imaging analysis tools, could be applied to this use-case, but are not yet validated for it. A primary qualitative investigation of stakeholder perceptions of such an AI-enabled decision tool is also absent. This multi-methods study aims to establish the safety and efficacy of an AI-enabled decision tool for nAMD treatment decisions and understand where on the clinical pathway it could sit and what factors are likely to influence its implementation.

Methods and analysis

Single-centre retrospective imaging and clinical data will be collected from nAMD clinic visits at an NHS teaching hospital ophthalmology service, including judgements of nAMD disease stability or activity made in real-world consultant-led-care. Dataset size will be set by a power calculation using the first 127 randomly sampled eligible clinic visits. An AI-enabled retinal segmentation tool and a rule-based decision tree will independently analyse imaging data to report nAMD stability or activity for each of these clinic visits. Independently, an external reading centre will receive both clinical and imaging data to generate an enhanced reference standard for each clinic visit. The non-inferiority of the relative negative predictive value of AI-enabled reports on disease activity relative to consultant-led-care judgements will then be tested. In parallel, approximately 40 semi-structured interviews will be conducted with key nAMD service stakeholders, including patients. Transcripts will be coded using a theoretical framework and thematic analysis will follow.

Ethics and dissemination

For peer review only - http://bmjopen.bmj.com/site/about/guidelines.xhtml

NHS Research Ethics Committee and UK Health Research Authority approvals are in place (21/NW/0138). Informed consent is planned for interview participants only. Written and oral dissemination is planned to public, clinical, academic and commercial stakeholders.

Keywords:

Age-related macular degeneration, non-inferiority study, AI validation, qualitative research, semi-structure interviews, implementation, fusion data

Strengths and limitations of this study

- This non-inferiority study examines an artificial intelligence (AI) use-case supported by patients and the public, with scope for improving the efficiency with which limited human resources are applied across the NHS and elsewhere.
- This AI use-case has a high degree of explainability as it relies on reviewable tissue segmentation and a simple rule-based decision tree which mimic widely used treatment paradigms.
- The multi-methods approach allows insights beyond efficacy alone to consider the effectiveness, mechanisms and system impacts of this complex intervention.
- Due to the pre-clinical nature of this study, stakeholder perspectives concerning this AI-use case will be hypothetical rather than from direct experience.
- As the non-inferiority study uses retrospective data only, the results will not reflect the challenges of real-time AI deployment which will require onward study.

For peer review only - http://bmjopen.bmj.com/site/about/guidelines.xhtml

Introduction

A threat to the efficacy of UK National Health Service (NHS) ophthalmology services is posed by insufficient ophthalmologist availability, in the face of a growing clinical need.[1, 2] Ophthalmology continued to incur more clinic appointments than any other NHS specialty through the Covid-19 pandemic, with age related macular degeneration (AMD) as the most common cause for these appointments.[2] AMD is the leading cause for certification of visual impairment in the UK and affects just over a quarter of Europeans aged over 60 to some extent.[3, 4] Only the disease course of a severe form of AMD, complicated by choroidal neovascularisation (nAMD), can benefit from current treatment and is thought to affect 1.4% of Europeans aged over 60.[3] Real-world data from a NHS centre showed that even before the impact of the Covid-19 pandemic, delay in delivering planned treatments for nAMD was common and associated with sight loss for the patients concerned.[5] This same NHS centre had recently displayed better nAMD visual outcomes than a mean of 12 large UK centres, suggesting the observed treatment delays are likely to be representative of the whole nation.[6] Since these observations, the Covid-19 pandemic has introduced additional nAMD treatment delays and avoidable sight loss, further developing the urgent need to augment clinical capacity for nAMD treatment.[7]

Clinical artificial intelligence (AI) is an increasing target of investment from government, industry and academia, often with the hope of increasing service capacity independent of the limits posed by clinician availability.[8-10] Ophthalmology is one of the most heavily researched specialties in AI medical image analysis with the non-contact infra-red based imaging modality, optical coherence tomography (OCT), accounting for much of this.[11] Retinal OCT scans form the substrate for several clinical AI tools, which anatomically segment the images to support various clinical decisions including triage and diagnosis.[12] The impact of these clinical AI functions on the efficacy and efficiency of primary to

For peer review only

secondary care referrals in ophthalmology are the subject of on-going prospective clinical studies.[13, 14] However, an even greater cumulative demand on ophthalmologists' time is making decisions about the treatment of nAMD within secondary care. Treatment takes the form of intravitreal injections (IVIs) of anti-vascular endothelial growth factor (anti-VEGF) agents which are recommended for variable periods of time and at varying intervals depending on changes in the retina observed through OCT.[15, 16] Whilst the decisions about treatments often involve ophthalmologists, the injections themselves are increasingly performed independently by specialised nurse practitioners and allied health professionals.[17, 18] Given the highly protocolised, OCT-dependent, nAMD treatment pathway and pre-existent clinical AI capable of independently quantifying the OCT features which inform that treatment, there is a clear opportunity for AI-enabled nAMD services to expand capacity and reduce avoidable real-world sight loss.[12, 16]

Despite this tension for change, clinical AI is yet to be implemented within routine NHS ophthalmic practice. This is at least partly due to the lack of evidence to forecast the effectiveness of clinical AI in the real-world setting and how it will interact with the various stakeholders and contexts which must host the technology.[19] Alongside other innovations such as portable OCT technology and community outreach treatment services, AI may enable even greater changes to the way in which macular services are delivered (Figure 1).[20, 21] The complexity derived from these interdependent technologies, contexts and stakeholders must be better understood if implementation is to optimise patient benefit from AI-enabled nAMD services. Given the years for which quantitative evidence of efficacy has existed for many clinical AI tools without subsequent real-world adoption, it may be that qualitative explorations of the complex implementation context are in fact the more urgent requirement.[11,22,23]

For peer review only - http://bmjopen.bmj.com/site/about/guidelines.xhtml

This single-centre multi-methods study aims to generate the necessary evidence by an external validation of the technology with real-world data and exploring the interdependent factors which will influence the implementation of AI-enabled nAMD services. In doing so, we aim to move beyond efficacy to consider intervention effectiveness and construct a theoretical basis to guide future implementation strategies.[22]

A quantitative study will be performed to test the non-inferiority of AI-enabled reports of nAMD disease activity against judgements made in consultant-led-care. In addition, a qualitative study based on semi-structured interviews with key stakeholders and patients will be conducted to explore where the clinical AI could sit within the nAMD care pathway and the factors likely to influence its implementation.

Methods and analysis

Quantitative methods

Sampling method

The electronic medical record (EMR) at Newcastle upon Tyne Hospitals NHS Foundation Trust (NuTH) will be searched to identify clinic visits where individuals received anti-VEGF IVI to treat nAMD. Patients at NuTH are treated under three different regimens dependent upon the length of their diagnosis and joint decisions between clinicians and patients: loading, treat-and-extend and pro-re-nata (Panel 1). The treatment intervals most commonly vary between 4 and 16 weeks. During the period through which data will be collected aflibercept and ranibizumab were the anti-VEGF treatments in use for nAMD.

For peer review only - http://bmjopen.bmj.com/site/about/guidelines.xhtml

Loading protocol: Starts at diagnosis of nAMD and consists of three anti-VEGF IVIs at four-week intervals (visits two and three do not include consultation or imaging), followed by IVIs at eight-week intervals, or less if signs of disease activity persist, for the remainder of the year.

Treat-and-extend (TEX) protocol: Starts one year after treatment initiation and dictates that the interval of anti-VEGF IVIs is increased in two-week increments until evidence of disease activity is noted at which point the treatment interval is reduced. If extension beyond a certain interval is noted to result in observable disease activity a (unspecified) number of times, then that interval ceases to be modified.

Pro-re-nata (PRN) protocol: Initiated as a joint decision for patients who appear to have little or no disease activity having been on one of the other two protocols. Here it is not assumed that an IVI will be given at each review, but only if evidence of disease activity is noted. The observation of returning disease activity may also lead to the return to one of the other two protocols.

Panel 1. Summary of neovascular age-related macular degeneration (nAMD) treatment at Newcastle upon Tyne Hospitals NHS Foundation Trust

IVI=Intravitreal Injection, VEGF = Vascular Endothelial Growth Factor.

Eligible visits from this dataset of 70,884 will be randomly selected for manual review of patient EMR files to screen against eligibility criteria (Panel 2). These criteria exclude clinical visits that took place before current treatment and OCT imaging protocols were established or after treatment decisions may have been influenced by the Covid-19 pandemic. They also exclude visits conducted under the PRN treatment protocol as all visits on this protocol meeting the inclusion criterion for a same day anti-VEGF IVI must have been

For peer review only - http://bmjopen.bmj.com/site/about/guidelines.xhtml

judged by the clinician to show disease activity and will therefore not be representative of PRN clinic visits generally. To maximise their relevance to the research question alongside feasibility and rigour within a complex real-world dataset, the eligibility criteria and systematic screening approach through which they will be applied was iteratively designed and trialled by authors with clinical, operational and statistical expertise inside and outside of NuTH (HDJH, SJT, PAK, KBa and DT) (Supplementary file 1). Having reached consensus on the eligibility criteria and the screening approach, a single researcher with 9 years of clinical experience at NuTH will perform data collection (HDJH), to support consistency of their application and ensure fluency with local clinical and digital practices.

For peer review only - http://bmjopen.bmj.com/site/about/guidelines.xhtml

Inclusion criteria

- **Eye diagnosed with nAMD**
- **One or more prior anti-VEGF IVI at NuTH**
- Co-located 25 slice, fovea centred OCT imaging available for both the included and prior visits
- Clinic visit note states intended IVI interval
- Clinic visit included same-day IVI

Exclusion criteria

- **Retinal diagnosis other than nAMD in included eye**
- Visit before 2016
- Visit during or after March 2020
- Visits conducted under the pro-re-nata treatment protocol

Panel 2. Eligibility criteria for patients (in bold) and clinic visits

nAMD = Neovascular age-related macular degeneration, IVI = Intravitreal injection, NuTH = Newcastle upon Tyne Hospitals, OCT = Optical coherence tomography.

Data collection and processing

For each included clinic visit the following data will be recorded to characterise the dataset:

- Anonym for the individual
- Eye laterality
- Gender
- Self-reported ethnicity
- Home address postal code stem
- Individual's age at that visit

For peer review only - http://bmjopen.bmj.com/site/about/guidelines.xhtml

The following will be recorded to send anonymised to Moorfields Reading Centre to generate a report of disease stability or activity to act as an enhanced reference standard for each visit. This information will also facilitate more meaningful post-hoc error analysis to explore the mechanisms of failure which the AI-enabled tool may exhibit. The findings from these analyses are a secondary outcome of the study and will help to delineate any groups of cases for which the tool's performance needs to be monitored and improved in further work, or for which only clinician judgements should be applied. This list was developed through additions to a proforma from a recent exemplar protocol:[24]

- OCT and visual acuity (VA) for that visit and the prior
- VA for the fellow eye at that visit
- Time since first nAMD treatment at that visit
- Total IVIs for nAMD in that eye up until that visit
- Observed interval since that eye's last IVI
- Presence or absence of evolving macular haemorrhage being recorded
- Time since increasing disease activity was last observed
- Treatment interval associated with that observation

The following data will also be recorded from each consultant-led-care visit to assess the primary and secondary outcomes and will not be sent to Moorfields Reading Centre:

- Judgement of disease activity or stability
- Planned interval to next IVI
- Professional group conducting the consultation
- Treatment protocol the visit was conducted under (Panel 1)

Separately, the present and prior pairs of OCT images relating to the same clinic visits will be transferred to Moorfields Eye Hospital NHS Foundation Trust for AI-enabled retinal

For peer review only - http://bmjopen.bmj.com/site/about/guidelines.xhtml

segmentation.[12] The differences in retinal tissue volumes will be used in a rule-based decision tree to produce an AI-enabled binary report of disease activity or stability for each included visit.

AI-enabled decision tool

The intervention to be tested on retrospective OCT imaging data is a deep learning tool with a U-net architecture with previously published details of training and validation.[12] It can produce volume quantification for the neurosensory retina, retinal pigment epithelium, fibrovascular pigment epithelium detachment, drusenoid pigment epithelium detachment, subretinal hyper-reflective material, subretinal fluid, intraretinal fluid, posterior hyaloid, epiretinal membrane and serous pigment epithelium detachment. An initial decision tree will report disease activity when the volumes of neurosensory retina, subretinal fluid or intraretinal fluid increase >5% between the current and prior visit OCTs.[25] This decision logic is based on a recent consensus from UK medical retina experts on treat-and-extend protocols for nAMD.[16] The exact tissue group contributors and decision thresholds for inter-visit changes in each of these tissue groups will be iterated upon using an embedded pilot dataset described further below. This binary output was preferred over a scalar recommendation of treatment interval to preserve the tools' value across different treatment protocols (Panel 1). The choice of a binary output will also help the generalisability of results, as the translation of OCT findings into treatment decisions varies internationally.

Outcomes measures

Over-treatment marginally increases the cumulative risk of IVI complications and the cost to the provider, but justifiably the main concern of patients and carers consulted in designing this study was sight loss through under-treatment.[5] Consequently, the probability of AI-enabled reports of disease stability being correct relative to judgements made in real-world consultant-led care has been taken as the most clinically relevant measure of diagnostic

For peer review only - http://bmjopen.bmj.com/site/about/guidelines.xhtml

accuracy. This has led to a non-inferiority design with the relative negative predictive value (NPV) as the primary outcome.

Secondary outcomes will be:

- A comparison of other standard diagnostic accuracy metrics between AI-enabled reports of disease activity and judgements from consultant-led-care accompanied by confusion matrices (Table 1)
- A comparison of the diagnostic accuracy of the five healthcare professional groups conducting consultations in consultant-led-care (nurses, optometrists, ophthalmology specialty trainees, medical retina sub-specialty fellows, medical retina sub-specialty consultants)
- A comparison of the treatment intervals recommended in real-world consultant-led care and the treatment intervals that would be derived from AI-enabled reports of disease activity given the treatment protocol
- A case-by-case exploration of false-positive and false-negative reports of disease activity from the AI-enabled decision tool and consultant-led care

Justification of study design and sample size

An enhanced reference standard is required to facilitate comparison between a potential AI-enabled nAMD service and the real-world gold standard, consultant-led-care. Moorfields Reading Centre has an international reputation and track record in meeting this need for prior studies and will receive imaging and clinical data for each case in the present study to generate an enhanced reference standard.[12, 13, 24] The binary decision under examination for each included visit is whether the data suggest disease stability or activity. This simplification of the scalar number of weeks between treatments or the three-option decision regarding treatment interval maintenance, extension or reduction is more broadly relevant to

For peer review only

real-world practice where clinician and patient preferences about how treatment intervals should be altered on account of disease activity vary (Panel 1).[16] It also lowers the risk of inappropriately labelling a decision made in consultant-led-care with subtle influences from patient or clinician preference as 'incorrect'. Whether binary judgements of disease activity (positives) or stability (negatives) from consultant-led-care and the AI-enabled decision tool are labelled as true or false will be decided by the independent judgement of the Moorfields Reading Centre (Table 1).

Table 1. Template confusion matrix showing the different possible classification of Artificial Intelligence (AI)-enabled reports of disease activity and judgements from consultant-led-care (CLC) for each eligible case

	Moorfields Reading Centre identifies disease stability (negative)	Moorfields Reading Centre identifies disease activity (positive)
AI identifies disease stability (negative)	AI True negative	AI False negative
AI identifies disease activity (positive)	AI False positive	AI True positive
CLC identifies disease stability (negative)	CLC True negative	CLC False negative
CLC identifies disease activity (positive)	CLC False positive	CLC True positive

Treatment decision data to support power calculations are unavailable for this novel use case. Considering a different use case, the deep learning model to be applied within the proposed AI-enabled decision tool has demonstrated equivalent or superior retinal diagnostic performance to consultant specialists. Relative to final real-world clinical diagnoses it produced an area under the ROC curve of 0.99 for retinal diseases including nAMD.[12] This performance was dependent on the same intermediate anatomical segmentation step that will form the basis for the AI-enabled decision tool for nAMD treatment proposed here. This has supported the feasibility of the current proposal but does not provide the level of certainty

For peer review only - http://bmjopen.bmj.com/site/about/guidelines.xhtml

required to perform a robust power calculation, which requires sufficiently accurate estimates of the NPV of both judgements from consultant-led-care and AI-enabled reports from paired data.[26] Therefore, a pilot dataset will be collected and sent for independent processing by Moorfields Reading Centre and the AI-enabled decision tool to supply these estimates. Prior work has established that for binary outcomes such as the one under study, little improvement is seen in estimate precision or bias by increasing the size of the pilot dataset above 100.[27] A preliminary review of 100 NuTH consultant-led-care nAMD clinic visits under loading or TEX treatment protocols found that 79 reported disease stability, and would be classed as negative cases which could contribute to estimating NPV. Given that about 79% of eligible visits are classed as negative the randomly sampled pilot dataset required to accrue 100 negative cases is expected to be around 127 cases (100 / 0.79), but the consultant-led-care judgement for each case will be reviewed as it is curated to ensure the pilot dataset contains 100 negative cases. The estimated NPV of AI-enabled reports and judgements from consultant-led-care will be derived from this pilot dataset and inform a power calculation. From this, the number of additional eligible cases required to be collected and processed by Moorfields Reading Centre and the AI-enabled decision tool to test the non-inferiority of AI-enabled report NPV relative to judgements from consultant-led-care will be established.[26] This power calculation will include a significance level $\alpha = 0.05$, a power $\beta = 0.90$ and a relative non-inferiority margin of $\delta = 0.90$. Although this non-inferiority margin is relative, it will be similar and no larger than an absolute equivalent. This allowed the application of 10% non-inferiority margins applied in comparable studies.[24, 28] In evaluating this non-inferiority margin it is helpful to remember that the least desirable outcome of a false negative would yield a two or four week delay for the next planned treatment rather than treatment cessation and that 22% of patients are estimated to experience more than four weeks of delay to treatment in a year in current consultant-led care.[5] As such the null

For peer review only - http://bmjopen.bmj.com/site/about/guidelines.xhtml

hypothesis will be rejected if the lower confidence limit for the relative NPV of AI-enabled reports is greater than 0.90.[26] This will be visually presented with 2-sided 95% confidence interval to explore the possibility of superiority with a threshold γ of 1/ δ; $\gamma = 1.11$ (Figure 2). Whilst the dataset will offer other important exploratory insights, the potential impact of the primary outcome on the translation of this AI-enabled decision tool and threat to the study's feasibility from ambitions outside this scope, has prevented any plans to proactively power the sample size for secondary outcomes.

Data analysis

The relative NPV of AI-enabled reports / judgements from consultant-led-care will be calculated with 95% confidence intervals to see if the inferiority, non-inferiority or superiority of AI-enabled reports of disease activity can be established (Table 1). For secondary outcomes, diagnostic accuracy statistics for each group will be reported descriptively with 95% confidence intervals for each group, along with confusion matrices. Clinical and imaging data from cases of false positives and false negatives of the AI-enabled reports will be reviewed by clinical members of the team, supported by the AI development team where necessary, to try to understand the mechanisms and potential consequences of AI-enabled decision tool failures. STARD reporting guidelines will be used alongside the STARD-AI extension if available at the time of writing.[29, 30]

For peer review only

Qualitative methods

Sampling

A recent systematic review outlined the importance of all stakeholder perspectives in understanding the interdependent factors that influence clinical AI implementation.[31,32] Despite this, the qualitative literature regarding clinical AI is dominated by healthcare professionals' perspectives and has particularly limited representation from carers.[31] Consequently, the present study will aim to explore perspective from all stakeholders. This

For peer review only - http://bmjopen.bmj.com/site/about/guidelines.xhtml

will include patients from the NuTH nAMD clinic, carers of patients from the same clinic, doctors, nurses, photographers and optometrists working in the clinics, primary care doctors and optometrists, hospital managers, relevant industry and charity sector professionals, and care commissioners. An approximate target of 40 participants has been set, but the targeting and scale of recruitment will be refined by themes and stakeholders identified in the data.

Data collection

Topic guides have been informed by the aforementioned systematic review and input from clinicians, academics, patients and lay members of the study team (Supplementary file 2). In their initial form these topic guides focus on:

- Stakeholders' experiences of the present macula service
- Their perceptions of clinical AI generally and specifically in recommending treatment intervals for nAMD
- Where this AI-enabled decision tool should be placed within the healthcare pathway and which cases or settings may not be appropriate
- Factors likely to affect the implementation of this AI-enabled decision tool
- How relationships between stakeholders and with the AI itself may change or develop

The topic guides will be used flexibly to guide semi-structure interviews and continuously iterated as data are analysed in parallel. Depending upon participant preference interviews may be conducted face-to-face or remotely by telephone or teleconferencing platforms. Interviews will be recorded and transcribed for text-based coding and analysis. Interviews will be delivered by the same researcher (HDJH) throughout, who is an ophthalmologist with lived experience of working in NuTH nAMD clinics and prior training and experience in qualitative research methods. Although patients with prior clinical contact with the interviewer will be ineligible for recruitment and the interviewer will be presented as a

For peer review only - http://bmjopen.bmj.com/site/about/guidelines.xhtml

researcher rather than a clinician, their status as a healthcare professional may emerge unintentionally and could influence data elicitation.[33] It is also true that some of the healthcare professional interviewees will have worked with the interviewer as a colleague in the past which is likely to influence data. Reflective journaling will be used to transparently report and manage these influences on data collection, analysis and interpretation.[34] Data will also be reviewed throughout the collection period by the wider study team, including lay representatives, to help identify and manage biases in sampling, data collection, analysis and interpretation.

Data analysis

The same researcher (HDJH) will familiarise themselves with data as it is collected and iteratively place data into a-priori codes and categories derived from a qualitative framework synthesis based on the Non-adoption, Abandonment, Scale-up, Spread and Sustainability framework (Figure 3).[35] These theoretical and empirical foundations will ensure data are structured in a way that accounts for the policy, organisational and practice level factors that influence implementation.[36] This will help to direct on going data collection, but also to rigorously curate data in preparation for thematic analysis.[37] Selection of a theoretical approach for data analysis will be informed by the data and systematic libraries of theoretical approaches used in clinical AI qualitative research and implementation research generally.[38, 39] COREQ reporting guidelines will be used.[40]

Patient and public involvement

The design of this protocol was informed by patient and carer members of the UK Macular Society and a National Institute for Health and Social Care Research (NIHR) funded lay consumer panel. These groups highlighted opportunities to improve current care by reducing

For peer review only

For peer review only - http://bmjopen.bmj.com/site/about/guidelines.xhtml

travel requirements for a population commonly affected by other health and social challenges and reducing the congestion of clinics and the time that patients spend there. The project continues to benefit from a study advisory group including a nAMD patient and carer who use the NuTH service and a reference group including four members of the public from across the UK. These individuals have helped design the topic guide and will continue to advise on sampling, data collection, analysis and interpretation.

Ethics and dissemination

The study has received NHS Research Ethics Committee and UK Health Research Authority approvals (21/NW/0138). Informed consent is planned for interview participants only. Though direct public access to data from the study is not supported, this ethics approval includes a means of sharing anonymised data following requests to the corresponding author and an application process. Dissemination is planned to clinical, academic and commercial stakeholders through peer reviewed conference presentation and open access journal publication. Public dissemination is planned through NIHR and macular society communication channels and a NIHR funded event in Newcastle upon Tyne for patients and their carers.

For peer review only - http://bmjopen.bmj.com/site/about/guidelines.xhtml

Contributors

HDJH led funding application and drafted the manuscript. All authors contributed to the study design. DT, KBr, KBa SJT, PAK and GM reviewed and revised the manuscript.

Funding

This study was part of a proposal funded by a National Institute for Health Research (NIHR) doctoral fellowship (NIHR301467). The funder had no role in study design, data collection, data analysis, data interpretation or manuscript writing.

Competing interests

The authors declare no competing interests.

References

1. The Royal College of Ophthalmologists, Work Force Census 2018.

2. NHS Digital, Hospital Outpatient Activity. 2021.

3. Li, J.Q., et al., Prevalence and incidence of age-related macular degeneration in Europe: a systematic review and meta-analysis. British Journal of Ophthalmology, 2020. 104(8): p. 1077.

4. The Royal National Institute for the Blind, Sight loss data tool. 2021.

5. Hogg, J., et al., The prevalence and impact of treatment delays in exudative age-related macular degeneration. Investigative Ophthalmology & Visual Science, 2021. 62(8): p. 2941-2941.

For peer review only

6. Talks, J.S., et al., Appropriateness of quality standards for meaningful intercentre comparisons of aflibercept service provision for neovascular age-related macular degeneration. Eye (Lond), 2017. 31(11): p. 1613-1620.

7. Arruabarrena, C., et al., Impact on visual acuity in neovascular age related macular degeneration (Namd) in europe due to covid-19 pandemic lockdown. Journal of Clinical Medicine, 2021. 10(15).

8. Muehlematter, U.J., et al., Approval of artificial intelligence and machine learning-based medical devices in the USA and Europe (2015–20): a comparative analysis. The Lancet Digital Health, 2021. 3(3): p. e195-e203.

9. World Health Organisation, Generating evidence for artificial intelligence-based medical devices: A framework for training validation and evaluation. 2021.

10. Guo, Y., et al., Artificial Intelligence in Health Care: Bibliometric Analysis. J Med Internet Res, 2020. 22(7): p. e18228.

11. Liu, X., et al., A comparison of deep learning performance against health-care professionals in detecting diseases from medical imaging: a systematic review and meta-analysis. The Lancet Digital Health, 2019. 1(6): p. e271-e297.

12. De Fauw, J., et al., Clinically applicable deep learning for diagnosis and referral in retinal disease. Nature Medicine, 2018. 24(9): p. 1342-1350.

13. Han J.E.D., et al., Teleophthalmology-enabled and artificial intelligence-ready referral pathway for community optometry referrals of retinal disease (HERMES): a Cluster Randomised Superiority Trial with a linked Diagnostic Accuracy Study—HERMES study report 1—study protocol; *BMJ Open* 2022;12:e055845.

For peer review only

For peer review only - http://bmjopen.bmj.com/site/about/guidelines.xhtml

14. Blandford, A., et al., Protocol for a qualitative study to explore acceptability, barriers and facilitators of the implementation of new teleophthalmology technologies between community optometry practices and hospital eye services. BMJ Open, 2022. 12(7): p. e060810.

15. National Institute for Health and Care Excellence, Age-related macular degeneration; NICE guideline 82. 2018.

16. Ross, A.H., et al., Recommendations by a UK expert panel on an aflibercept treat-and-extend pathway for the treatment of neovascular age-related macular degeneration. Eye, 2020. 34(10): p. 1825-1834.

17. Bolme, S., et al., Task shifting of intraocular injections from physicians to nurses: a randomized single-masked noninferiority study. Acta Ophthalmol, 2020. 98(2): p. 139-144.

18. Hasan, H., S. Mamtora, and N. Shah, Setting up a successful nurse-led intravitreal injections service: Pearls from Swindon. British Journal of Nursing, 2020. 29(20): p. 1178-1185.

19. González-Gonzalo, C., et al., Trustworthy AI: Closing the gap between development and integration of AI systems in ophthalmic practice. Progress in Retinal and Eye Research, 2021: p. 101034.

20. von der Burchard, C., et al., Self-examination low-cost full-field OCT (SELFF-OCT) for patients with various macular diseases. Graefe's Archive for Clinical and Experimental Ophthalmology, 2021. 259(6): p. 1503-1511.

21. Amoaku, W., et al., Providing a safe and effective intravitreal treatment service: strategies for service delivery. Clinical Ophthalmology, 2020. 14: p. 1315-1328.

For peer review only

For peer review only - http://bmjopen.bmj.com/site/about/guidelines.xhtml

22. Skivington, K., et al., A new framework for developing and evaluating complex interventions: update of Medical Research Council guidance. BMJ, 2021. 374: p. n2061.

23. Maniatopoulos, G., Hunter, D. J., Erskine, J., & Hudson, B. (2020). Implementing the New Care Models in the NHS: Reconfiguring the Multilevel Nature of Context to Make It Happen. In *Transitions and Boundaries in the Coordination and Reform of Health Services* (pp. 3-27). Palgrave Macmillan, Cham.

24. Learoyd, A.E., et al., FENETRE study: quality-assured follow-up of quiescent neovascular age-related macular degeneration by non-medical practitioners: study protocol and statistical analysis plan for a randomised controlled trial. BMJ Open, 2021. 11(5): p. e049411.

25. Moraes, G., et al., Quantitative Analysis of OCT for Neovascular Age-Related Macular Degeneration Using Deep Learning. Ophthalmology, 2021. 128(5): p. 693-705.

26. Moskowitz, C.S. and M.S. Pepe, Comparing the predictive values of diagnostic tests: sample size and analysis for paired study designs. Clinical Trials, 2006. 3(3): p. 272-279.

27. Teare, M.D., et al., Sample size requirements to estimate key design parameters from external pilot randomised controlled trials: a simulation study. Trials, 2014. 15: p. 264.

28. Reeves, B.C., et al., Effectiveness of Community versus Hospital Eye Service follow-up for patients with neovascular age-related macular degeneration with quiescent disease (ECHoES): a virtual non-inferiority trial. BMJ Open, 2016. 6(7): p. e010685.

29. Bossuyt, P.M., et al., STARD 2015: an updated list of essential items for reporting diagnostic accuracy studies. Bmj, 2015. 351: p. h5527.

For peer review only - http://bmjopen.bmj.com/site/about/guidelines.xhtml

30. Sounderajah, V., et al., Developing a reporting guideline for artificial intelligence-centred diagnostic test accuracy studies: the STARD-AI protocol. BMJ Open, 2021. 11(6): p. e047709.

31. Al-Zubaidy, M., et al., Stakeholder Perspectives on Clinical Decision Support Tools to Inform Clinical Artificial Intelligence Implementation: Protocol for a Framework Synthesis for Qualitative Evidence. JMIR Res Protoc, 2022. 11(4): p. e33145.

32. Hogg HDJ, Al-Zubaidy M, Technology Enhanced Macular Services Study Reference Group et al. Multi-stakeholder perspectives of clinical artificial intelligence implementation: systematic review of qualitative evidence. J Med Internet Res (forthcoming). doi:10.2196/39742

33. Richards, H. and C. Emslie, The 'doctor' or the 'girl from the University'? Considering the influence of professional roles on qualitative interviewing. Family Practice, 2000. 17(1): p. 71-75.

34. Ortlipp, M., Keeping and using reflective journals in the qualitative research process. The Qualitative Report, 2008. 13(4): p. 695-705.

35. Greenhalgh, T., et al., Beyond Adoption: A New Framework for Theorizing and Evaluating Nonadoption, Abandonment, and Challenges to the Scale-Up, Spread, and Sustainability of Health and Care Technologies. J Med Internet Res, 2017. 19(11): p. e367.

36. Maniatopoulos, G., et al., Moving beyond local practice: Reconfiguring the adoption of a breast cancer diagnostic technology. Social Science & Medicine, 2015. 131: p. 98-106.

37. Braun, V., et al., Using thematic analysis in psychology. Qualitative Research in Psychology, 2006. 3(2): p. 77-101.

For peer review only

38. Rabin, B., et al. Dissemination and Implementation Models in Health Research and Practice 2021; Available from: https://dissemination-implementation.org.

39. Hogg, H.D.J., et al., Implementing Implementation Science; A Bibliometric Study of Qualitative Research in Clinical Artificial Intelligence. Research Square, 2022.

40. Tong, A., P. Sainsbury, and J. Craig, Consolidated criteria for reporting qualitative research (COREQ): a 32-item checklist for interviews and focus groups. Int J Qual Health Care, 2007. 19(6): p. 349-57.

For peer review only

Legends to figures

Figure 1. Process map of current 'one-stop' macular clinic visits for a patient

Some steps have the potential to be substituted by AI-enabled treatment decisions based on OCT imaging (thick black line), some could be relocated to primary care optometry (thin black line) and some could also be decentralised using community outreach treatment services (dashed black line).

Figure 2. Forest plot template for the relative negative predictive value (NPV) of artificial intelligence (AI)-enabled reports of neovascular age-related macular degeneration (nAMD) disease activity versus judgements from consultant-led-care relative to an enhanced reference standard from Moorfields Reading Centre

The non-inferiority and superiority margins are marked by dashed vertical lines on a logarithmic scale at 0.90 and 1.11 respectively. Potential outcomes for the non-inferiority test include scenario (a) AI-enabled reports are inferior to judgements from consultant-led-care;

For peer review only - http://bmjopen.bmj.com/site/about/guidelines.xhtml

(b) Non-inferiority of AI-enabled reports to judgements from consultant-led-care is not demonstrated; (c) AI-enabled reports are non-inferior to judgements from consultant-led-care; (d) AI-enabled reports are non-inferior to judgements from consultant-led-care but not superior; (e) AI-enabled reports are superior to judgements from consultant-led-care.

Figure 3. Schematic of the Non-adoption, Abandonment, Scale-up, Spread and Sustainability (NASSS) framework [35]

For peer review only

For peer review only - http://bmjopen.bmj.com/site/about/guidelines.xhtml

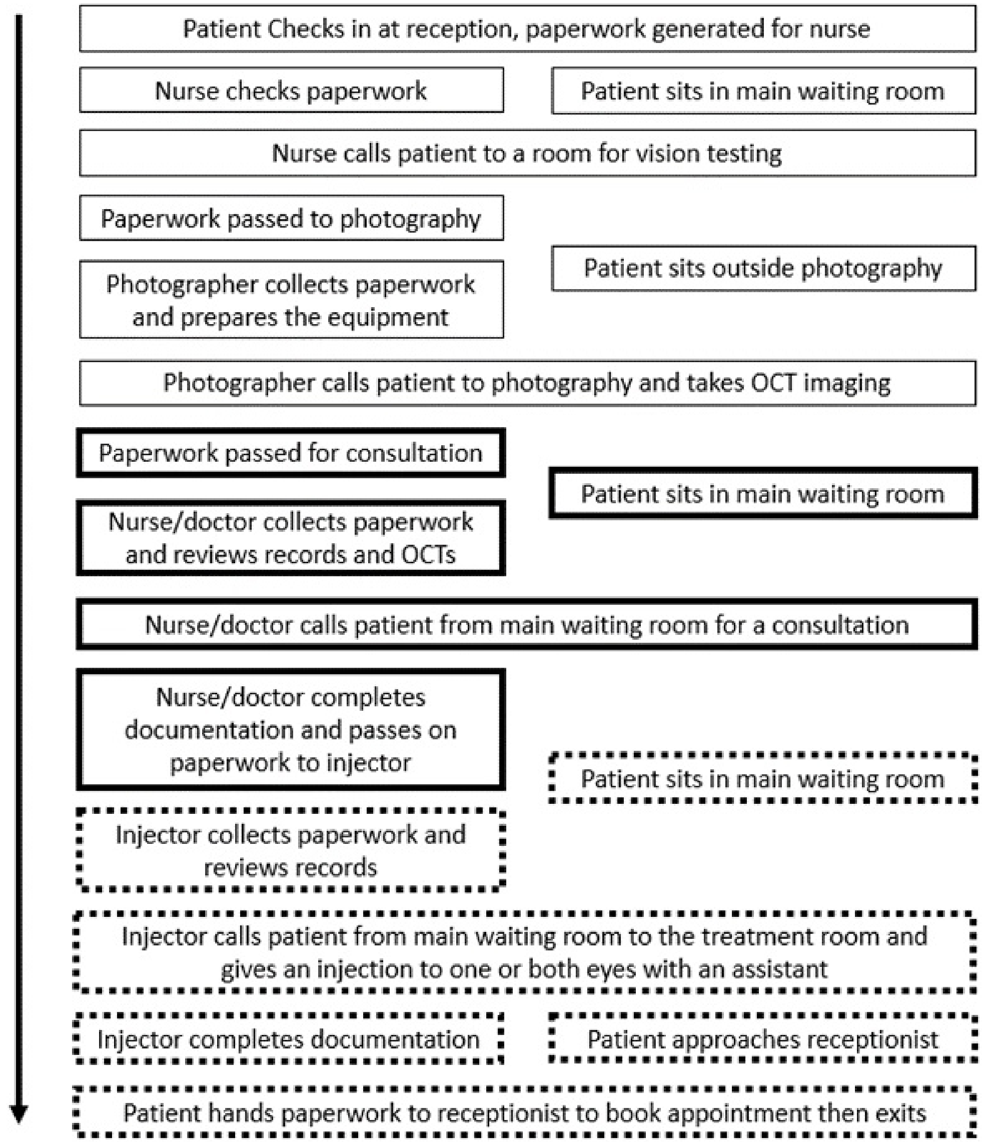

Process map of current 'one-stop' macular clinic visits for a patient. Some steps have the potential to be substituted by AI-enabled treatment decisions based on OCT imaging (thick black line), some could be relocated to primary care optometry (thin black line) and some could also be decentralised using community outreach treatment services (dashed black line).

106x123mm (144 x 144 DPI)

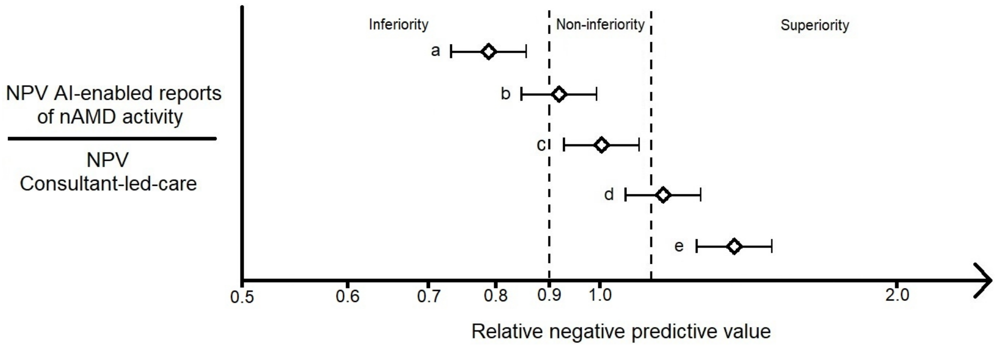

Forest plot template for the relative negative predictive value (NPV) of artificial intelligence (AI)-enabled reports of neovascular age-related macular degeneration (nAMD) disease activity versus judgements from consultant-led-care relative to an enhanced reference standard from Moorfields Reading Centre. The non-inferiority and superiority margins are marked by dashed vertical lines on a logarithmic scale at 0.90 and 1.11 respectively. Potential outcomes for the non-inferiority test include scenario (a) AI-enabled reports are inferior to judgements from consultant-led-care; (b) Non-inferiority of AI-enabled reports to judgements from consultant-led-care is not demonstrated; (c) AI-enabled reports are non-inferior to judgements from consultant-led-care; (d) AI-enabled reports are non-inferior to judgements from consultant-led-care but not superior; (e) AI-enabled reports are superior to judgements from consultant-led-care.

240x83mm (144 x 144 DPI)

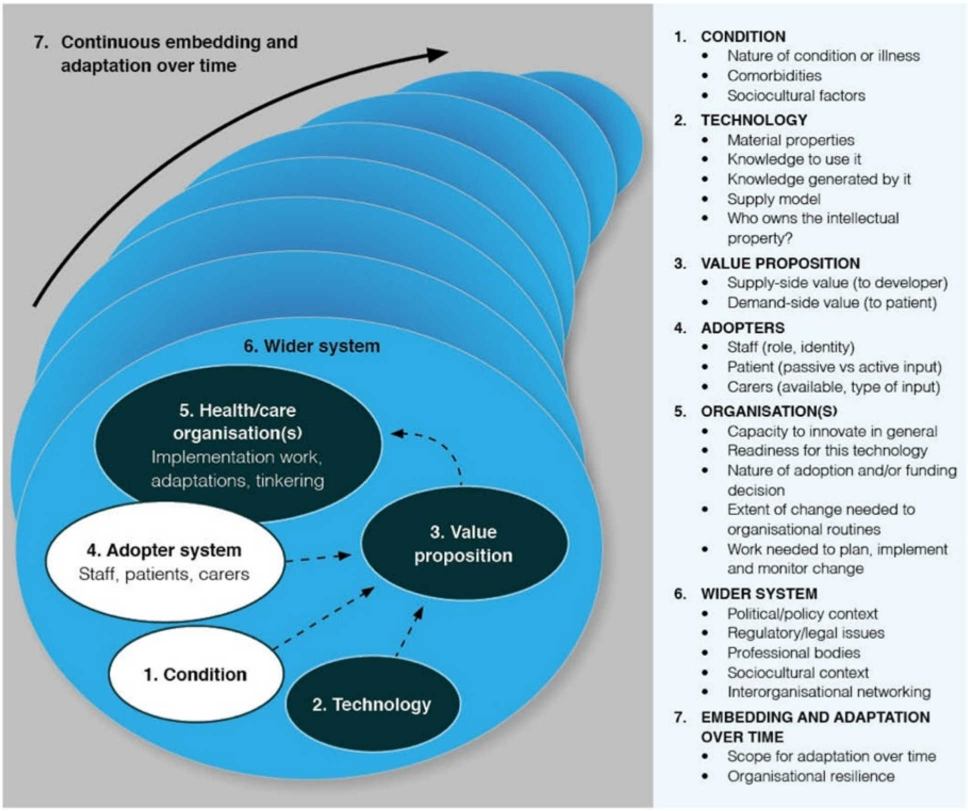

Figure 3. Schematic of the Non-adoption, Abandonment, Scale-up, Spread and Sustainability (NASSS) framework. [35]

165x137mm (144 x 144 DPI)

Systematic sampling strategy

Step	Question	Action if... Yes	Action if... No
**1**	Does this eye have no retinal diagnosis beside AMD or is it enrolled in a study?	Go to step 2	Reject this patient
**2**	Is this visit more than 10 weeks after the eye's first IVI?	Go to step 3	Switch to the next visit, go to step 2
**3**	Does this visit involve anti-VEGF treatment for nAMD?	Go to step 4	Switch to the next visit, go to step 2
**4**	Is this visit conducted under the loading or TEX protocols?	Go to step 5	Switch to the next visit, go to step 2
**5**	Are the VAs of interest free from the influence of other interventions?	Go to step 6	Switch to the next visit, go to step 2
**6**	Does this visit have an accompanying consultation recorded?	Go to step 7	Switch to the next visit, go to step 2
**7**	Is the treatment interval stated?	Go to step 8	Switch to the next visit, go to step 2
**8**	Is there a VA available for this visit and the prior?	Go to step 9	Switch to the next visit, go to step 2
**9**	Are there co-located 25 slice fovea-centred OCTs available for this visit and the prior?	Collect data	Switch to the next visit, go to step 2

AMD = Age-related macular degeneration, IVI = Intravitreal Injection, VEGF = Vascular Endothelial Growth Factor, nAMD = neovascular Age-related macular degeneration, TEX = Treat and Extend, VA = Visual Acuity, OCT = Optical Coherence Tomography

For peer review only

Patient topic guide

Research question

What place do stakeholders in macular services think artificial intelligence ought to take in nAMD care pathways and what are the barriers and facilitators to its adoption there?

Topics and example questions

Current pathway

1. What parts of the current service do you like most?
2. What changes would you like to see in the current service?
3. In the current service you meet lots of team members for different parts of your care. Which bits of face-to-face or written contact do you find most valuable?
4. Have you had any 'injection only' or virtual clinic visits, where a consultation isn't part of the visit? If so, how do you find them?

Initial impressions of clinical AI

1. As you know, I'm interested in the idea of technology in healthcare. What are your first thoughts and feelings when I mention artificial intelligence in healthcare?
2. When I'm talking about AI here, I mean a type of technology designed to take in some information and make a decision about it on its own. Specifically, I'm talking about a kind of AI we have, which can look at your eye photos on its own and see when your eye next needs an injection without help from a nurse or doctor. What use could you imagine for AI like that?
3. What kind of down-sides or difficulties do you think there might be in using that kind of AI in the clinic?

Pathway placement

1. Who do you think the best person to be responsible for the AI would be?
2. What kind of interactions would you like to have with doctors if artificial intelligence is brought in?
3. Where would you like to have your eye photos taken?
4. Where would you like to have your injections given?

Relationships with the tool and others

1. Some people might feel a bit uncomfortable about letting AI take some of the responsibility for their treatment planning. What kind of things might help you trust AI like this?
2. With this kind of AI, you might be able to see how and why it makes its treatment decisions for you. How would you feel about that?
3. How might it change the way friends and family support you in managing your eye disease?
4. Who would you want to be able to access the AI's decision making?
5. How do you think bringing this AI into the service would affect your relationships with different members of the care team?

Closing

1. Thanks very much for so many helpful insights. Is there anything else we haven't talked about that seems important about using AI in macular degeneration clinics?
2. We're planning on talking to hospital doctors and nurses, opticians and managers but do you think there are other people's perspectives we should be hearing?

For peer review only
